# A novel approach to understanding bird communities using informed diversity estimates at local and regional scales in northern California and southern Oregon

**DOI:** 10.1002/ece3.5008

**Published:** 2019-03-15

**Authors:** Jared D. Wolfe, John D. Alexander, Jaime L. Stephens, C. John Ralph

**Affiliations:** ^1^ School of Forest Resources and Environmental Sciences Michigan Technological University Houghton Michigan; ^2^ Klamath Bird Observatory Ashland Oregon; ^3^ U.S.D.A. Forest Service Pacific Southwest Research Station Arcata California

**Keywords:** assemblage, community, community similarity, DCA, informed indices, species richness

## Abstract

Assessment and preservation of biodiversity has been a central theme of conservation biology since the discipline's inception. However, when diversity estimates are based purely on measures of presence–absence, or even abundance, they do not directly assess in what way focal habitats support the life history needs of individual species making up biological communities. Here, we move beyond naïve measures of occurrence and introduce the concept of “informed diversity” indices which scale estimates of avian species richness and community assemblage by two critical phases of their life cycle: breeding and molt. We tested the validity of the “informed diversity” concept using bird capture data from multiple locations in northern California and southern Oregon to examine patterns of species richness among breeding, molting, and naïve (based solely on occurrence) bird communities at the landscape and local scales using linear regression, community similarity indices, and a Detrended Correspondence Analysis (DCA). At the landscape scale, we found a striking pattern of increased species richness for breeding, molting, and naïve bird communities further inland and at higher elevations throughout the study area. At the local scale, we found that some sites with species‐rich naïve communities were in fact species‐poor when informed by breeding status, indicating that naïve richness may mask more biologically meaningful patterns of diversity. We suggest that land managers use informed diversity estimates instead of naïve measures of diversity to identify ecologically valuable wildlife habitat.

## 1 INTRODUCTION

Identification of ecologically valuable habitats for birds is often based on measures of species richness and evenness (collectively referred to as “diversity” hereafter; Gaston & Spicer, 2013) whereby areas with more biologically meaningful resources are assumed to host heightened levels of diversity. Yet the process of distinguishing valuable habitat based on measures of diversity may be flawed due to inherent difficulties associated with detecting every species within a community (Iknayan, Tingley, Furnas, & Beissinger, 2014; MacKenzie, Nichols, Hines, Knutson, & Franklin, 2003). Additionally, the mobility of many bird species can confound the relationship between estimates of diversity and habitat value. For example, simple vagaries of geography can funnel migrating and dispersing birds onto desolate peninsulas or shorelines, thereby breaking linkages between measures of avian diversity and habitat value (e.g., DeSante, 1983; Vladimir, 1998). At fine spatial scales, such as these, avian mobility and ecological traps can result in birds occupying inferior habitats, leading to erroneous conclusions about a location's capacity to support a diverse bird community (e.g., Mänd, Tilgar, & Lõhmus, 2005; Gilroy, Anderson, Vickery, Grice, & Sutherland, 2011). The territorial nature of many birds further limits our capacity to prioritize habitat conservation with measures of diversity. For instance, territoriality can result in ideal despotic distributions where dominant individuals force subordinates into marginal habitat, thereby confounding relationships between diversity and habitat quality (Johnson, 2007). Clearly, the development of additional methodologies that moderate biases associated with these intrinsic processes and behaviors represent a critical step toward informed conservation planning.

In this vein, many studies have focused exclusively on breeding birds to assess the influence of habitat fragmentation, forest openings, urbanization, trophic cascades, and other perturbations on measures of avian diversity (Germaine, Vessey, & Capen, 1997; Jokimäki, Suhonen, Jokimäki‐Kaisanlahti, & Carbó‐Ramírez, 2014; Lynch & Whigham, 1984; McShea & Rappole, 2000). By focusing on breeding birds, researchers aim to eliminate the influence of floaters or other individuals that are not directly reliant on resources within a given habitat during an energetically taxing phase of the avian life cycle. Although vitally important, breeding is but a single facet of the avian life cycle and is often structured in relation to the timing of another vital life cycle event—molt (Pyle et al. 1997; Howell et al. 2010). Relative to research focused on breeding, the energetically costly act of molt is an underrepresented topic in the literature, as has been pointed out by Wolfe and Pyle (2012) and, to our knowledge, has not been used to inform measures of bird diversity. We strongly feel that the diversity of molting bird communities, similar to breeding communities, should be considered when identifying valuable habitats because the timing of molt in temperate birds has likely been adapted to occur during periods of elevated food resources (Rohwer, Rohwer, & Barry, 2008; Pyle et al., 2009; Howell et al. 2010). As such, birds exhibit remarkable strategies, such as altitudinal and long‐distance migration to intermediate stops before proceeding to the wintering grounds, to acquire the necessary food resources to complete molt (Pyle et al., 2009; Rohwer et al., 2008; Wiegardt, Barton, & Wolfe, 2017). Thus, many bird species rely on separate locations to undergo breeding and molting events resulting in a patchwork of habitats with varying ecological value for breeding and molting bird communities. It is imperative to identify and conserve valuable habitats for birds throughout the entire annual cycle, not just during breeding periods.

In the western United States, for example, at least some forest‐dwelling birds will nest in closed canopy forest and then make small‐scale movements to meadows and riparian areas with abundant food to molt during the postbreeding period (Wilkerson & Siegel 2002, Wiegardt et al., 2017). Changes in habitat use between the breeding and molting season result in differences between breeding and molting bird diversity at local scales. Furthermore, changes in more general habitat characteristics at broader spatial scales, such as variation in elevation and distance from climate‐moderating effects of coastlines, may also have considerable influence on the value of habitat and subsequent diversity of breeding and molting bird communities across the landscape. Additionally, patterns of avian diversity at landscape scales may in part be driven by seasonal availability of insects and fruit, both important food resources for birds, at higher elevations later in the breeding season (Tanaka & Tanaka, 1982).

In this study, we compared naïve diversity measures (i.e., diversity measures based solely on occurrence) with informed diversity measures (i.e., those informed by breeding and molting activity) across multiple spatial scales. Specifically, we examined breeding, molting, and naïve bird community assemblages, species richness, and evenness to identify areas of conservation value by addressing three questions: (a) can variation in breeding, molting, and naïve bird diversity be explained by geospatial elements of elevation and distance inland from the coast? (b) At local scales, does species richness and community assemblage differ when considering breeding, molting, and naïve bird communities, suggesting that the value of habitats change with respect to avian life cycle phenology? (c) Does accounting for differences in species richness and community assemblage across the avian life cycle provide a more holistic framework to assess the value of habitat? To answer these questions, we examined patterns of bird diversity based on 22 years of capture data from 25 banding stations dispersed across northern California and southern Oregon. Our study represents the first investigation that prioritizes habitats based on the diversity of both breeding and molting birds at the local and landscape scale.

## 2 MATERIALS AND METHODS

**Figure 1:**
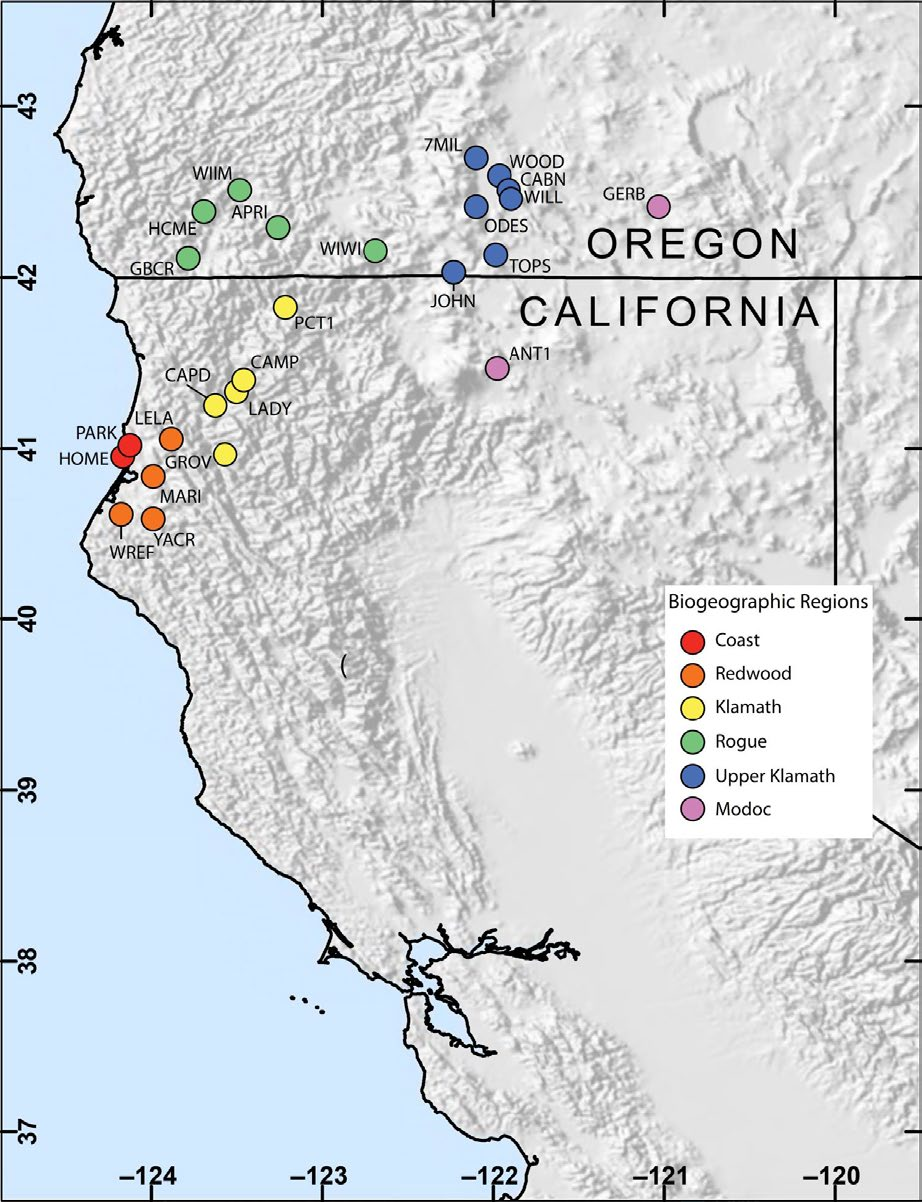
Map of the study area in northern California and southern Oregon. Each of the 25 bird capture stations included in this study is identified by circles and further aggregated into biogeographic regions

Bird capture data were collected between 1992 and 2010 at 25 different capture stations throughout northern California and southern Oregon (referred to hereafter as the “study area” *see* Figure 1) (Alexander, 2011; Alexander, Ralph, Hollinger, & Hogoboom, 2004). Data were collected under USFWS banding permit #9082 and with approval of the USDA Forest Service. Each station had between 10 and 22 years of data; all data are archived in the Avian Knowledge Network database (Iliff et al., 2009). Stations occurred across a diversity of elevations and distances from the coast and fell within eight biogeographic regions (Table 1). Although environmentally diverse, each banding station occurred adjacent to or within a forested, riparian, and/or meadow landscape. One year of effort consisted of a single banding day at least every 7–10 days, from mid‐May until mid‐October; although a few stations were operated two to three times per week. A single banding day was 5 or 6 hours of effort, starting within 15 minutes of sunrise. Each banding station had between 10–15 12 × 3 m, 36 mm mesh mist nets. Given that banding efforts were focused on sampling landbirds that occur in these habitats, we removed unrepresentative species that are not commonly captured in these habitats (e.g., raptors, owls, waterbirds, swallows and others, *see* Supporting information Appendix S1). Additionally, we removed all hatching‐year birds to reduce the influence of transients. Banding data were then subdivided into three bird communities based on data taken during capture (following protocols in Ralph, Geupel, Pyle, Martin, & DeSante, 1993): first, a breeding bird community was comprised of individuals captured with a smooth, vascularized or wrinkled brood patch, or a medium or large cloacal protuberance. Second, a molting bird community was comprised of individuals captured undergoing symmetrical flight feather molt. And third, the naïve bird community was comprised of all captured individuals irrespective of breeding and molting status.

**Table 1:** Effort and location of the 25 capture stations in northern California and southern Oregon

Station	Effort (years)	Inland (m)	Elevation (m)	Region	Latitude	Longitude
PARK	16	550	6	Coast	40.89476	−124.143
HOME	22	771	6	Coast	40.89047	−124.142
CAPD	15	40,679	112	Klamath	41.26045	−123.606
CAMP	11	43,829	119	Klamath	41.29576	−123.559
LADY	13	44,764	111	Klamath	41.2928	−123.548
GROV	16	53,161	1,258	Klamath	40.95627	−123.486
PCT1	16	78,664	409	Klamath	41.84304	−123.211
ANT1	17	177,356	1,656	Modoc	41.49405	−121.94
GERB	11	261,547	1,481	Modoc	42.17325	−121.042
WREF	16	7,164	16	Redwood	40.78209	−124.122
MARI	13	14,582	33	Redwood	40.84754	−123.988
LELA	10	17,885	16	Redwood	40.53986	−124.142
YACR	10	23,273	49	Redwood	40.56016	−124.059
HCME	18	61,362	864	Rogue	42.38509	−123.668
GBCR	11	67,654	656	Rogue	42.14984	−123.418
WIIM	18	75,214	246	Rogue	42.49077	−123.48
APRI	11	87,084	352	Rogue	42.29374	−123.235
WIWI	11	127,485	556	Rogue	42.19888	−122.691
JOHN	15	165,568	1,559	Upper Klamath	42.24798	−122.234
TOPS	14	172,659	964	Upper Klamath	42.02612	−122.101
ODES	16	183,697	1,263	Upper Klamath	42.43048	−122.062
CABN	16	184,326	1,264	Upper Klamath	42.49696	−122.08
7MIL	15	190,427	1,279	Upper Klamath	42.70501	−122.074
WOOD	15	198,900	1,263	Upper Klamath	42.58763	−121.933
WILL	14	207,471	1,276	Upper Klamath	42.65635	−121.854

To determine differences in species richness relative to breeding, molting, and naïve bird communities across stations, we used sample‐based rarefaction at each station for captures between the months of May and October, and then extrapolated each rarefaction curve to 40 years of effort in program EstimateS (Colwell 2005). In addition to species richness estimates, we also generated Shannon*–*Wiener index values (H) to examine measures of evenness for breeding, molting, and naïve bird communities at each station. We subsequently z‐transformed estimates of species richness and evenness, relative to breeding, molting, and naïve richness and evenness, to make estimates more comparable. For example, positive and negative species richness estimates (z‐transformed) are associated with stations that were found to have relatively more or less species, respectively.

To examine relationships between geographic attributes and species richness relative to breeding, molting, and naïve bird communities, we employed a series of linear regressions where the response variable was estimated species richness (obtained through rarefaction, see above) for breeding, molting, and naïve birds at each station, and explanatory variables were either elevation (linear and quadratic) or distance inland from coast (linear and quadratic) for each station. Both elevation and distance inland are colinear and were, therefore, not included within the same model. Top models were selected using corrected Akaike Information Criterion (AICc) values (Table 2). Adjusted *R* ^2^, beta estimates and associated 95% confidence intervals for each top model were also examined, and presented here, for exploratory purposes. All regression models were formulated and evaluated in program R. To determine if patterns of species richness were correlated between breeding, molting, and naïve bird communities, we regressed estimates of species richness for breeding, molting, and naïve bird communities from each station against each other and used adjusted *R* ^2^, beta estimates, and associated 95% confidence intervals to evaluate correlations.

**Table 2:** Model rankings for linear and quadratic (indicated by the superscript “2”) regression analyses for naïve, breeding, and molting bird communities captured at 25 stations in northern California and southern Oregon

Model	AIC_c_	∆_AICc_	w_AICc_	*k*
Naïve				
Distance inland	186.64	0.00	0.47	2
Distance inland^2^	187.99	1.36	0.24	3
Elevation^2^	188.07	1.44	0.23	3
Elevation	191.01	4.37	0.05	2
Null	193.03	6.40	0.02	1
Breeding				
Distance inland	183.62	0.00	0.47	2
Distance inland^2^	186.20	0.41	0.38	2
Elevation^2^	185.80	2.18	0.16	3
Elevation	187.65	4.04	0.06	3
Null	191.40	7.79	0.01	1
Molting				
Distance inland	179.55	0.00	0.47	2
Distance inland^2^	181.54	2.00	0.17	3
Elevation	182.61	3.06	0.10	2
Elevation^2^	183.21	3.67	0.07	3
Null	189.00	9.46	0.00	1

To assess community similarity and difference between stations, we used Chao's abundance‐based Jaccard community similarity indices. The formula used for Chao‐Jaccard abundance‐based similarity indices are based on Chao, Chazdon, Colwell, and Shen (2005) and described by Colwell (2005) where *Q_1_ *is the frequency of uniques, *Q_2_ *the frequency of duplicates:

$$v\hat{a}r\left({\hat{S}}_{\text{chao2}}\right)=Q_{2}+\left[\frac{1}{2}{\left(\frac{Q_{1}}{Q_{2}}\right)}^{2}+{\left(\frac{Q_{1}}{Q_{2}}\right)}^{3}+\frac{1}{4}{\left(\frac{Q_{1}}{Q_{2}}\right)}^{4}\right]$$

and:

$${\hat{S}}_{\text{chao2}}={\text{S}}_{\text{observations}}+\frac{{\text{Q}}_{1}^{2}}{{\text{Q}}_{2}}$$

According to Colwell (2005), Chao's abundance‐based Jaccard community similarity indices are based on the probability that two randomly chosen individuals, one from each of the two samples, both belong to species shared by the two samples (but not necessarily to the same shared species; Chao et al., 2005; Colwell, 2005). These methods reduce biases associated with traditional community similarity methodologies (Chao et al., 2005; Colwell, 2005). All Chao's abundance‐based Jaccard indices for each pair of stations were subsequently z‐transformed (allowing comparisons across breeding, molting, naïve bird communities) and arranged by elevation to examine patterns of similarity across different altitudes (Supporting information Appendix S2).

To visualize and further explore differences between community assemblages for breeding, molting, and naïve bird communities relative to station and biogeographic region, we used package Vegan in Program R (Dixon, 2003; R Core Team, 2013) to separately ordinate bird communities at each station, standardized by taking the quotient of the total number of individuals captured by the number of years of effort for each station, via a Detrended Correspondence Analysis (DCA). The relative strengths of the DCA axes were given as eigenvalues, and the relative importance of each axis in explaining variance in the dataset was determined by dividing the value for that axis by the sum of the four eigenvalues produced by the DCA (Supporting information Appendix S3). We examined each station for differences relative to the other stations via vector fitting analyses and a permutation test using 1,000 iterations in package Vegan (Supporting information Appendix S4). We conducted three separate DCA analyses for breeding, molting, and naïve bird communities. Further, we constructed convex hull polygons for breeding, molting, and naïve bird communities for each biogeographic region that hosted four or more capture stations (Upper Klamath, Rogue, Klamath, and Redwood, Table 1, Supporting information Appendix S5).

## 3 RESULTS

**Figure 2:**
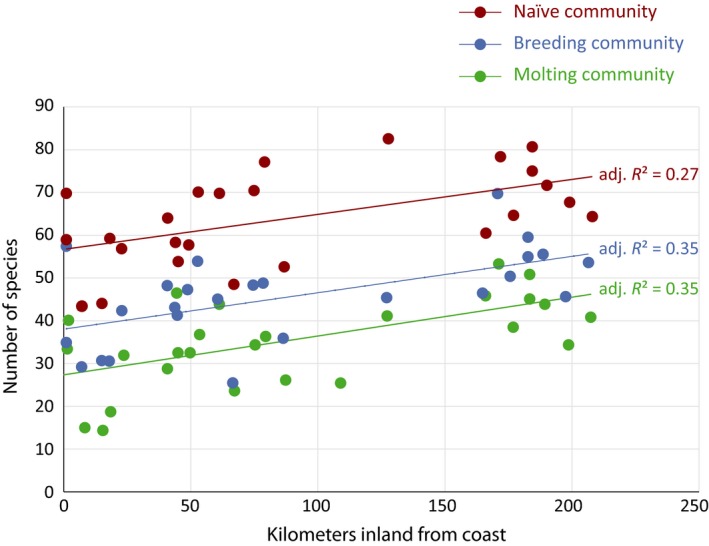
Visualizations of the top linear regressions for estimated species richness of breeding, molting, and naïve communities captured from May to October, correlated with distance from coast

**Figure 3:**
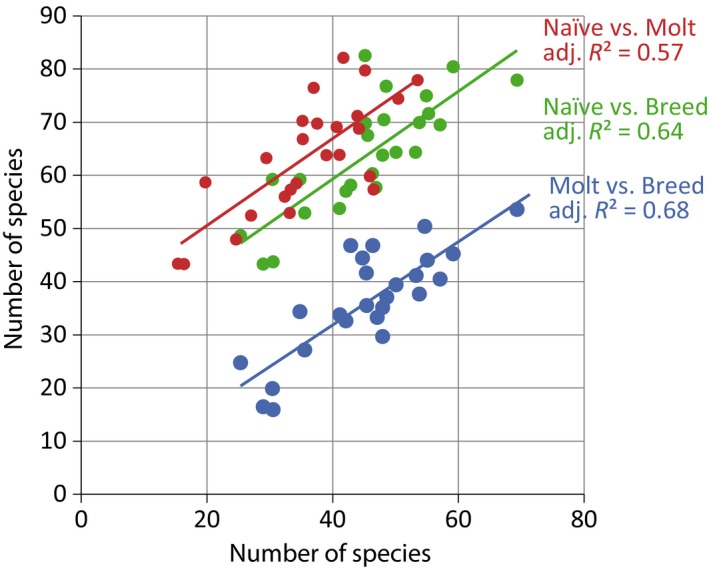
Visualizations of linear regressions for estimated species richness of breeding, molting, and naïve bird communities captured in southern Oregon and northern California

We first examined patterns of breeding and molting species richness at the landscape scale. Specifically, we regressed breeding, molting, and naïve bird species richness with distance inland (linear and quadratic) and elevation (linear and quadratic) and found that breeding (β = 0.08, 95% CI = 0.04, 0.13), molting (β = 0.09, 95% CI = 0.04, 0.13), and naïve (β = 0.08, 95% CI = 0.03, 0.13) bird communities were more species rich further inland (Figure 2). To determine if patterns of species richness were correlated between breeding, molting, and naïve bird communities, we regressed estimates of species richness for breeding, molting, and naïve bird communities from each station against each other and found positive relationships (Figure 3). Specifically, species richness was positively correlated between breeding and molting communities (β = 0.86, 95% CI = 0.62, 1.10), breeding and naïve communities (β = 0.77, 95% CI = 0.53, 1.01), and molting and naïve communities (β = 0.82, 95% CI = 0.53, 1.11).

We also examined patterns of species richness and evenness at the local scale to identify stations that switched from being relatively species rich in one life cycle stage (i.e., breeding) to being relatively depauperate in another stage (i.e., molting; Supporting information Appendix S6). We identified six stations (24% of the total) where richness was *under‐ *and *over*represented with regards to breeding or molting species richness (Table 3). For example, CAPD, GERB, and WIIM all had above average species richness when examining breeding communities, and below average species richness when examining molting communities. Conversely, CAMP, HCME, and WIWI all had below average species richness when examining breeding communities, and above average species richness when examining molting communities. Our analysis also identified five stations (20% of the total) that exhibited above average species richness and evenness irrespective of breeding, molting, and naïve communities: CABN, HOME, ODES, PCT1, and WILL (Table 3). Of these, CABN had the highest species richness with regards to the breeding community and ODES had the highest species richness with regards to the molting community. We used Chao's abundance‐based Jaccard community similarity indices to examine similarities between breeding, molting, and naïve communities at each station. Our results suggested that stations at comparable elevations were most similar. In general, stations were most similar when considering naïve and most dissimilar when considering molting bird communities.

**Table 3:** Z‐transformed diversity statistics for 25 capture stations in northern California and southern Oregon

Station	Naïve			Breeding			Molting		
	Rarefaction (extrapolated 40 years)	Chao I (10‐year)	Shannon (10‐year)	Rarefaction (extrapolated 40 years)	Chao I (10‐year)	Shannon (10‐year)	Rarefaction (extrapolated 40 years)	Chao I (10‐year)	Shannon (10‐year)
7MIL	0.70	0.59	0.95	0.93	0.87	1.08	0.78	0.73	−0.16
ANT1	0.04	−0.45	0.50	0.44	0.58	0.82	0.29	0.68	0.70
APRI	−1.02	−0.67	0.53	−0.94	−0.99	0.41	−0.91	−0.96	0.77
CABN	1.53	1.21	1.38	1.31	1.32	0.97	0.90	1.06	1.41
**CAMP**	**−0.55**	−0.09	0.20	**−0.25**	−0.84	−0.45	**1.06**	1.48	0.45
**CAPD**	**−0.01**	−0.34	0.59	**0.24**	0.12	0.30	**−0.66**	−0.68	0.66
GBCR	−1.42	−0.95	−1.07	−1.93	−1.78	−0.56	−1.16	−1.18	−0.23
**GERB**	**−0.57**	−0.39	0.66	**0.15**	0.80	0.97	**−0.28**	0.05	−0.12
GROV	0.55	0.45	0.30	0.78	−0.33	1.31	0.14	−0.53	−1.37
**HCME**	**0.51**	−0.02	0.30	**−0.06**	−0.04	1.08	**0.82**	−0.06	−0.09
HOME	0.52	0.68	0.59	1.11	1.28	0.19	0.45	0.09	1.02
**JOHN**	**−0.33**	0.23	0.43	**0.07**	0.01	0.71	**1.01**	0.87	0.63
LADY	−0.94	−0.55	−0.16	−0.42	−0.77	−0.45	−0.30	0.25	−0.05
LELA	−0.45	−0.99	−1.66	−1.44	−1.14	−1.49	−1.64	−1.30	−0.77
MARI	−1.87	−2.00	−1.86	−1.43	−1.45	−1.45	−2.04	−1.71	−1.73
ODES	1.02	1.15	1.22	0.88	1.23	1.20	1.44	1.23	1.91
PARK	−0.44	−0.77	−1.07	−1.03	−1.24	−1.23	−0.21	−0.42	−1.62
PCT1	1.21	0.84	0.56	0.29	0.25	0.30	0.06	0.28	0.91
TOPS	1.32	1.56	−0.12	2.28	1.69	−0.11	1.74	1.84	−0.73
**WIIM**	**0.61**	0.43	0.14	**0.25**	0.75	−0.22	**−0.09**	0.28	0.27
WILL	0.04	0.46	0.82	0.75	0.75	0.82	0.49	0.21	0.88
**WIWI**	**1.73**	1.55	0.86	**−0.03**	0.14	−0.07	**0.56**	0.83	−0.84
**WOOD**	**0.34**	0.65	−0.81	**−0.01**	0.34	−2.05	**−0.09**	−0.27	0.05
WREF	−1.90	−2.46	−2.44	−1.57	−1.63	−1.87	−1.99	−2.21	−2.02
YACR	−0.64	−0.12	−0.84	−0.32	0.09	−0.22	−0.36	−0.53	0.09

Note

Bolded stations indicate a switch from negative to positive value, or vice versa, with regards to rarefaction estimates of species richness across naïve, breeding, and molting bird communities. Rarefaction estimates are based on extrapolations to 40 years while Chao I and Shannon estimates are based on 10 years of data.

Finally, we visualized community assemblages of breeding, molting, and naïve bird communities at each station using a Detrended Correspondence Analysis (DCA). The first two axes of the DCA for breeding, molting, and naïve bird communities explained approximately 67%, 72%, and 69% of the cumulative variation within the data, respectively (Supporting information Appendix S3). The DCA permutation and vector fitting analyses indicated that naïve and breeding bird communities at stations nearer the coast and at lower elevations (Coastal, Redwood and Klamath regions) were more different when compared to bird communities at higher elevations and farther inland (Supporting information Appendix S4). In general, community assemblage appeared to depend on whether we considered breeding, molting, or naïve bird communities. The DCA convex hull polygons encompassing regions with four or more stations suggested that the Redwood region was most different exhibiting no overlap with any of the other regions irrespective of life cycle phase (Supporting information Appendix S5). Conversely, the Rogue and Klamath regions were most similar with broadly overlapping convex hull polygons across breeding, molting, and naïve communities (Supporting information Appendix S5).

## 4 DISCUSSION

We used a long‐term banding dataset to examine patterns of species richness and assemblage for breeding, molting, and naïve bird communities at the landscape and local scales. Our study provided four important findings: (a) patterns of species richness among breeding, molting, and naïve bird communities varied predictably across the landscape with more species occurring inland rather than nearer the coastline. (b) Irrespective of some stations being nearer to each other, local conditions often resulted in starkly different estimates of species richness. (c) The relative number of species often differed between stations when considering breeding, molting, and naïve bird communities, suggesting that habitat value changes with respect to avian life cycle phenology. (d) Informed estimates of species richness provide a more holistic framework to assess the habitat value for birds across their entire life cycle.

At the landscape scale, we found a pattern of increased species richness for breeding, molting, and naïve bird communities further inland throughout the study area (Figure 2). We believe our inland sites were more species rich because birds tended to move inland and to higher elevations to exploit seasonally abundant food resources during the breeding and molting seasons. More specifically, as the study area became increasingly dry during the breeding and molting seasons, we believe moisture refugia, like mountain meadows and riparian areas, become increasingly important to bird communities reliant on arthropod food resources to successfully complete nesting and molt. The assertion of inland and upslope movements in our study area has been observed by Wiegardt et al. (2017) who found more molting and breeding Wilson's Warblers at higher and lower elevations, respectively, suggesting that dry conditions facilitate upward movements of some birds. Our results indicate that putative inland and high elevation habitats, such as mountain meadows in western forests, support diverse breeding and molting bird communities during dry summer and fall months.

In addition to identifying patterns of diversity at the landscape scale, informed indices provide a powerful metric to assess the relative value of local habitats across breeding and molting seasons. For example, we examined the distribution of species rich and poor stations with regard to breeding, molting, and naïve bird communities. We identified four stations with relatively high breeding and molting bird species richness: CABN, ODES, 7MIL, and TOPS. Each of these stations occurred at high elevations, hosted multilayered forest next to water features, and were situated near the Modoc plateau desert. Thus, these sites provide habitat features critical to sustaining rich bird communities near desert‐like conditions and serve as climatic refugia throughout multiple phases of the avian life cycle; we suggest these areas exhibit high conservation value for provisioning the resources necessary to sustain diverse bird communities throughout most of their annual cycle. Conversely, WREF and MARI, both within the Redwood region exhibited the lowest species richness with regards to breeding and molting communities. However, WREF did host unique breeding and molting bird community assemblages as well as 63% of all Pacific Wrens captured in the study, thereby illustrating the importance of conserving diversity at different scales; WREF is seemingly less important when considering diversity at the local (alpha and beta) scale but may be critical in maintaining diversity at broader (gamma) spatial scales (Table S1).

**Figure 4:**
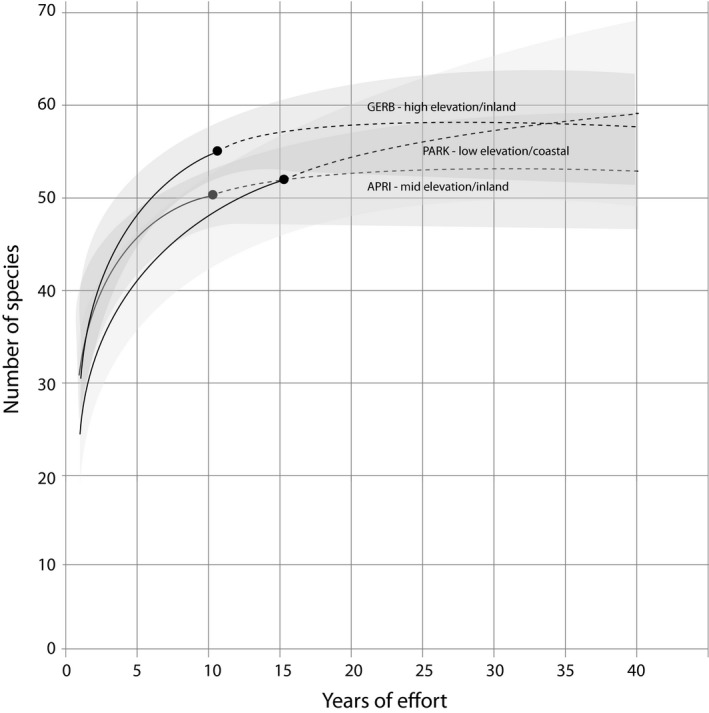
Example rarefaction curves for naïve (total) bird community captured at three stations that differ in distance from coast and elevation. Solid lines indicate years of actual effort and dotted lines indicate extrapolations to 40 years of effort. Shaded areas indicate 95% confidence intervals

**Figure 5:**
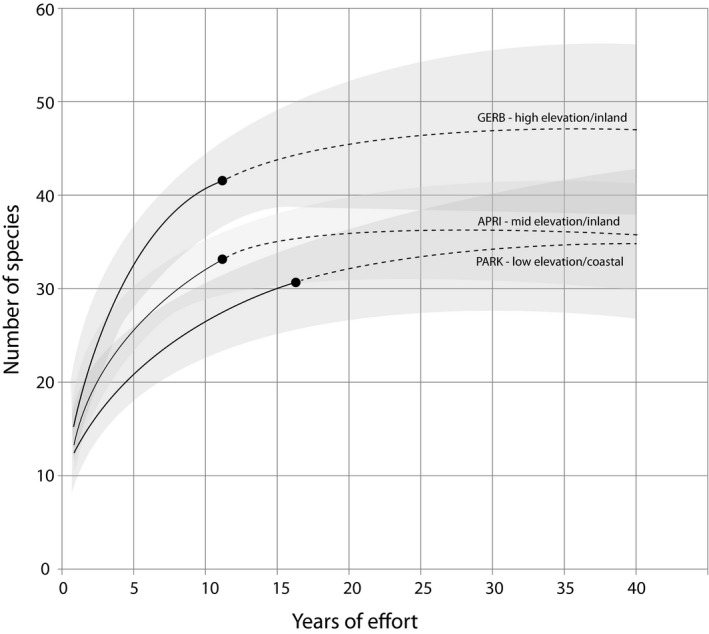
Example rarefaction curves for all landbird species captured in breeding condition at three stations that differ in distance from coast and elevation. Solid lines indicate years of actual effort and dotted lines indicate extrapolations to 40 years of effort. Shaded areas indicate 95% confidence intervals

Differences in naïve and breeding bird species richness suggest that informed diversity estimates provided more precision when determining the ecological value of habitat. For example, we found little difference in species richness between stations such as PARK and GERB when considering naïve bird communities; however, GERB was found to be more species rich than PARK when examining breeding bird communities (Figures 4, 5). Differences in naïve and breeding bird species richness between PARK and GERB demonstrate the value of capturing birds—we can't assume a site has value by presence alone—otherwise, GERB might be overlooked. The same is true for important habitats for molting birds. We found three stations (CAMP, HCME, and WIWI) that exhibited above average species richness when considering molting birds, and below average species richness when considering breeding bird communities (Table 3). Relative to breeding birds, we also found greater differences in molting bird community assemblage at capture stations throughout the study area and believe these differences demonstrate how the ecological value of locations can vary relative to the changing habitat requirements of birds as they end breeding and begin to molt.

**Figure 6:**
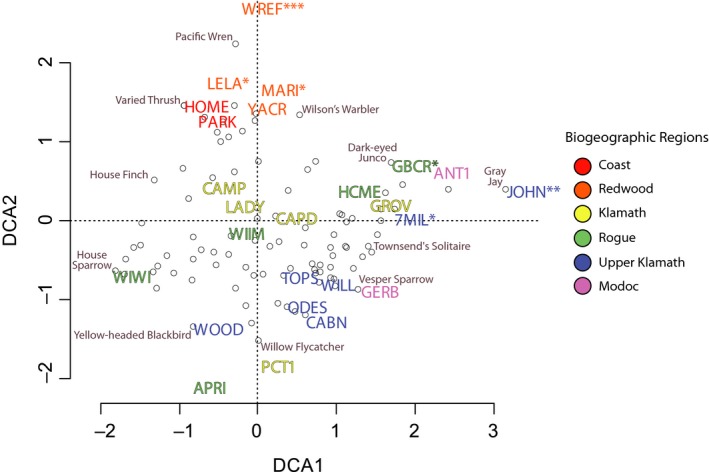
Detrended Correspondence Analysis (DCA) ordination for breeding bird communities based on the abundance of captured individuals per species from May to October at 25 stations (denoted by four‐letter code). Each station's biogeographic region is signified in the ordination by a unique color. Hollow dots represent individual bird species. Permutation tests were conducted to identify those bird communities found at each station that varied significantly relative to others, where *, **, and *** denote *p*‐values less than 0.05, 0.001, and 0.0001, respectively

**Figure 7:**
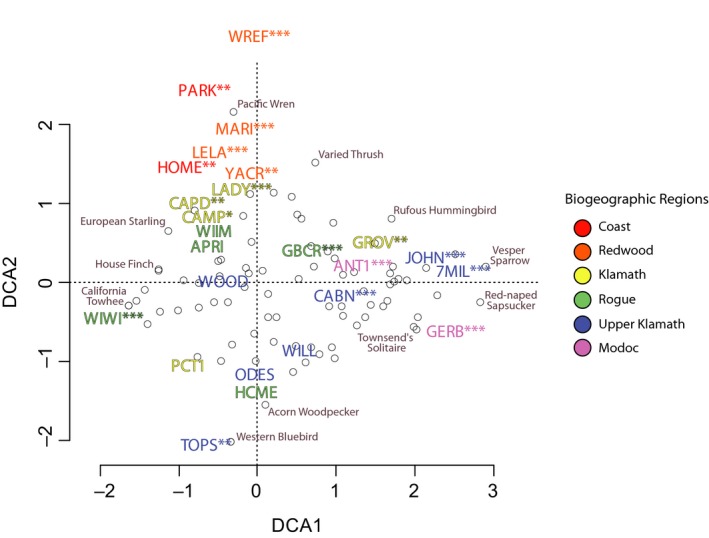
Detrended Correspondence Analysis (DCA) ordination for molting bird communities based on the abundance of captured individuals per species from May to October at the 25 stations (denoted by four‐letter code). Each station's biogeographic region is signified in the ordination by a unique color Hollow dots represent individual bird species. Permutation tests were conducted to identify those bird communities found at each station that varied significantly relative to others, where *, **, and *** denote p‐values less than 0.05, 0.001, and 0.0001, respectively

**Figure 8:**
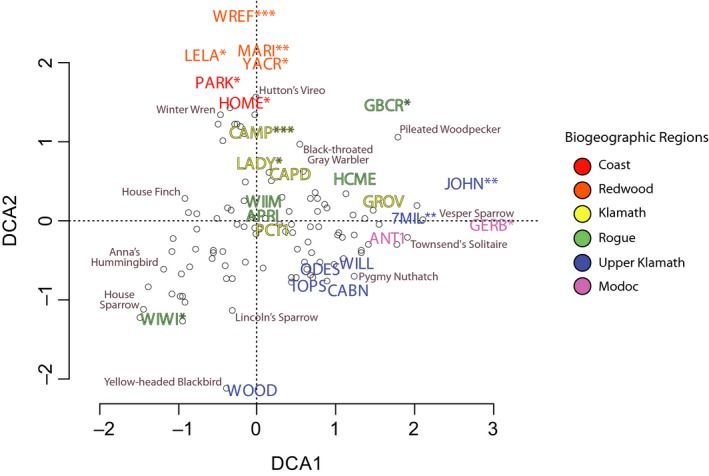
Detrended Correspondence Analysis (DCA) ordination for naïve (total) bird communities based on the abundance of captured individuals per species from May to October at the 25 stations (denoted by four‐letter code). Each station's biogeographic region is signified in the ordination by a unique color. Hollow dots represent individual bird species. Permutation tests were conducted to identify those bird communities found at each station that varied significantly relative to others, where *, **, and *** denote p‐values less than 0.05, 0.001, and 0.0001, respectively

Our ordination and community similarity analyses of breeding, molting, and naïve community assemblage found that stations in the Redwood region tended to host more unique breeding bird communities relative to more inland sites (Figure 6). Adjacent to the Redwood region, we detected differences in estimates of species richness between stations in the Coastal region that were separated by only a few kilometers (*see* PARK and HOME stations; Table 3). In general, the Redwood region was relatively species poor with distinct assemblages (Figures 4, 7, 8); the four stations within the Redwood region exhibited below average species richness with regards to breeding, molting, and naïve communities when compared to all other stations (Table 3). Such differences suggest that few species are adapted to breeding and molting in the relatively homogenous redwood forests. When compared to breeding bird communities, molting birds showed heightened variation in community assemblage across the study area. For example, permutation and vector fitting analyses suggested that molting bird communities at individual stations were often more different relative to each other irrespective of elevation, kilometers inland, or region (Figure 7). These findings were supported by generally lower estimates of community similarity between stations when considering molting bird communities, relative to naïve and breeding communities (Supporting information Appendix S2). Our results suggest that molting bird community assemblage exhibit more variation and turnover, relative to breeding communities, across the study area.

Here, we demonstrated that local variation in breeding, molting, and naïve bird species richness manifest as predictable patterns across the landscape. Further, we found informed measures of diversity can be used to better assess the value of habitat for conservation action. These results demonstrate that informed diversity indices represent a powerful tool to measure the ecological value of habitat across the entire avian life cycle. Researchers should use tools such as informed diversity indices to assess habitat value throughout the entire avian life cycle to promote a more holistic approach to habitat management and conservation.

## AUTHOR CONTRIBUTIONS

Dr. Jared Wolfe and Dr. John Alexander contributed to this manuscript during its conception, preliminary analysis, data collection, analysis, writing, and revisions. Dr. C. J. Ralph contributed to this manuscript during conception, data collection, writing, and revisions. Jaime Stephens contributed to this manuscript during the data collection, writing, and revisions phases of this manuscript.

## Data Availability

All data used in this study are archived in the Avian Knowledge Network Northwest (available here: http://www.avianknowledgenorthwest.net/data-management/banding-data-archive)

## References

[ece35008-bib-0001] Alexander, J. D. (2011). Advancing landbird conservation on western federally managed lands with management‐and policy‐relevant science. Prescott, AZ: Prescott College.

[ece35008-bib-0002] Alexander, J. D. , Ralph, C. J. , Hollinger, K. , & Hogoboom, B. (2004). Using a wide‐scale landbird monitoring network to determine landbird distribution and productivity in the Klamath Bioregion In Mergenthaler K. L., Williams J. E., & Jules E. S., (Eds.), (pp. 33–41). Proceedings of the Second Conference on Klamath‐Siskiyou Ecology. Cave Junction, OR: Siskiyou Field Institute.

[ece35008-bib-0003] Chao, A. , Chazdon, R. L. , Colwell, R. K. , & Shen, T. J. (2005). A new statistical approach for assessing similarity of species composition with incidence and abundance data. Ecology Letters, 8(2), 148–159. 10.1111/j.1461-0248.2004.00707.x

[ece35008-bib-0004] Colwell, R. K. (2005). EstimateS: Statistical estimation of species richness and shared species from samples. Version, 7(5), 2005. User's Guide and Application published at: http://purloclcorg/estimates.

[ece35008-bib-0005] R Core Team , (2013). R: A language and environment for statistical computing. Vienna, Austria: R Foundation for Statistical Computing.

[ece35008-bib-0006] DeSante, D. F. (1983). Annual variability in the abundance of migrant landbirds on Southeast Farallon Island, California. The Auk, 95, 826–852.

[ece35008-bib-0007] Dixon, P. (2003). VEGAN, a package of R functions for community ecology. Journal of Vegetation Science, 14(6), 927–930. 10.1111/j.1654-1103.2003.tb02228.x

[ece35008-bib-0008] Gaston, K. J. , & Spicer, J. I. (2013). Biodiversity: An introduction. Oxford: Blackwell Publishing.

[ece35008-bib-0009] Germaine, S. S. , Vessey, S. H. , & Capen, D. E. (1997). Effects of small forest openings on the breeding bird community in a Vermont hardwood forest. Condor, 708–718. 10.2307/1370482

[ece35008-bib-0010] Gilroy, J. J. , Anderson, G. Q. A. , Vickery, J. A. , Grice, P. V. , & Sutherland, W. J. (2011). Identifying mismatches between habitat selection and habitat quality in a ground‐nesting farmland bird. Animal Conservation, 14(6), 620–629. 10.1111/j.1469-1795.2011.00480.x

[ece35008-bib-0011] Howell, S. N. (2010). Molt in North American Birds. Boston, MA: Houghton Mifflin Harcourt.

[ece35008-bib-0012] Iknayan, K. J. , Tingley, M. W. , Furnas, B. J. , & Beissinger, S. R. (2014). Detecting diversity: Emerging methods to estimate species diversity. Trends in Ecology & Evolution, 29(2), 97–106. 24315534 10.1016/j.tree.2013.10.012

[ece35008-bib-0013] Iliff, M. , Salas, L. , Inzunza, E. R. , Ballard, G. , Lepage, D. , & Kelling, S. (2009). The Avian Knowledge Network: a partnership to organize, analyze, and visualize bird observation data for education, conservation, research, and land management. In Proceedings of the Fourth International Partners in Flight Conference: Tundra to Tropics. (Eds T Rich)

[ece35008-bib-0014] Johnson, M. D. (2007). Measuring habitat quality: A review. The Condor, 109(3), 489–504. 10.1650/8347.1

[ece35008-bib-0015] Jokimäki, J. , Suhonen, J. , Jokimäki‐Kaisanlahti, M. L. , & Carbó‐Ramírez, P. (2014). Effects of urbanization on breeding birds in European towns: Impacts of species traits. Urban Ecosystems, 19, 4431–13. 10.1007/s11252-014-0423-7

[ece35008-bib-0016] Lynch, J. F. , & Whigham, D. F. (1984). Effects of forest fragmentation on breeding bird communities in Maryland, USA. Biological Conservation, 28(4), 287–324. 10.1016/0006-3207(84)90039-9

[ece35008-bib-0017] MacKenzie, D. I. , Nichols, J. D. , Hines, J. E. , Knutson, M. G. , & Franklin, A. B. (2003). Estimating site occupancy, colonization, and local extinction when a species is detected imperfectly. Ecology, 84(8), 2200–2207. 10.1890/02-3090

[ece35008-bib-0018] Mänd, R. , Tilgar, V. , & Lõhmus, A. (2005). Providing nest boxes for hole-nesting birds–Does habitat matter? Biodiversity & Conservation, 14(8), 1823–1840.

[ece35008-bib-0019] McShea, W. J. , & Rappole, J. H. (2000). Managing the abundance and diversity of breeding bird populations through manipulation of deer populations. Conservation Biology, 14(4), 1161–1170. 10.1046/j.1523-1739.2000.99210.x

[ece35008-bib-0021] Pyle, P. (1997). Identification guide to North American birds: Part I Columbidae to Ploceidae. Bolinas, CA: Slate Creek Press.

[ece35008-bib-0022] Pyle, P. , Leitner, W. A. , Lozano‐Angulo, L. , Avilez‐Teran, F. , Swanson, H. , Limón, E. G. , & Chambers, M. K. (2009). Temporal, spatial, and annual variation in the occurrence of molt‐migrant passerines in the Mexican monsoon region. The Condor, 111(4), 583–590. 10.1525/cond.2009.090085

[ece35008-bib-0023] Ralph, C. J. , Geupel, G. R. , Pyle, P. , Martin, T. E. , & DeSante, D. F. (1993). Handbook of field methods for monitoring landbirds. USDA Forest Service General Technical Report PSW-GTR-144-www. Berkley, CA: Pacific Southwest Research Station.

[ece35008-bib-0024] Rohwer, V. G. , Rohwer, S. , & Barry, J. H. (2008). Molt scheduling of western Neotropical migrants and up‐slope movement of Cassin's Vireo. The Condor, 110(2), 365–370. 10.1525/cond.2008.8321

[ece35008-bib-0027] Tanaka, L. K. , & Tanaka, S. K. (1982). Rainfall and seasonal changes in arthropod abundance on a tropical oceanic island. Biotropica, 14, 114–123. 10.2307/2387740

[ece35008-bib-0028] Vladimir, A. (1998). Age structure of passerine migrants at the eastern Baltic coast: The analysis of the “coastal effect”. Ornis Svecica, 8, 171–178.

[ece35008-bib-0029] Wiegardt, K. A. , Barton, D. C. , & Wolfe, J. D. (2017). Upslope molt migration of the Wilson's Warbler in the Klamath‐Siskiyou Bioregion. Journal of Ornithology, 88, 47–52. 10.1111/jofo.12185

[ece35008-bib-0030] Wilkerson, R. L. , & Siegel, R. B. (2002). Establishing a southern sierra meadows important bird area: Results from meadow surveys at Stanislaus, Sierra, and Sequoia National Forests, and Yosemite and Sequoia/Kings Canyon National Parks. Point Reyes Station, California: The Institute for Bird Populations.

[ece35008-bib-0031] Wolfe, J. D. , & Pyle, P. (2012). Progress in our understanding of molt patterns in Central American and Caribbean landbirds. Ornitologıa Neotropical, 23, 169–176.

