# Supplementary material for: A novel approach to understanding bird communities using informed diversity estimates at local and regional scales in northern California and southern Oregon
Source: Ecol Evol. 2019 Mar 15;9(8):4431–42. doi: 10.1002/ece3.5008 (PMC6476868; doi:10.1002/ece3.5008)
Supplement: Supplementary file 1 [file ECE3-9-4431-s001.docx]

Figure S1. Results from the Chao’s abundance-based Jaccard community similarity analysis assessing breeding bird communities at the 25 capture stations (z-transformed values and arranged by elevation). Lower (-) and higher (+) values represent dissimilar and similar bird communities, respectively.


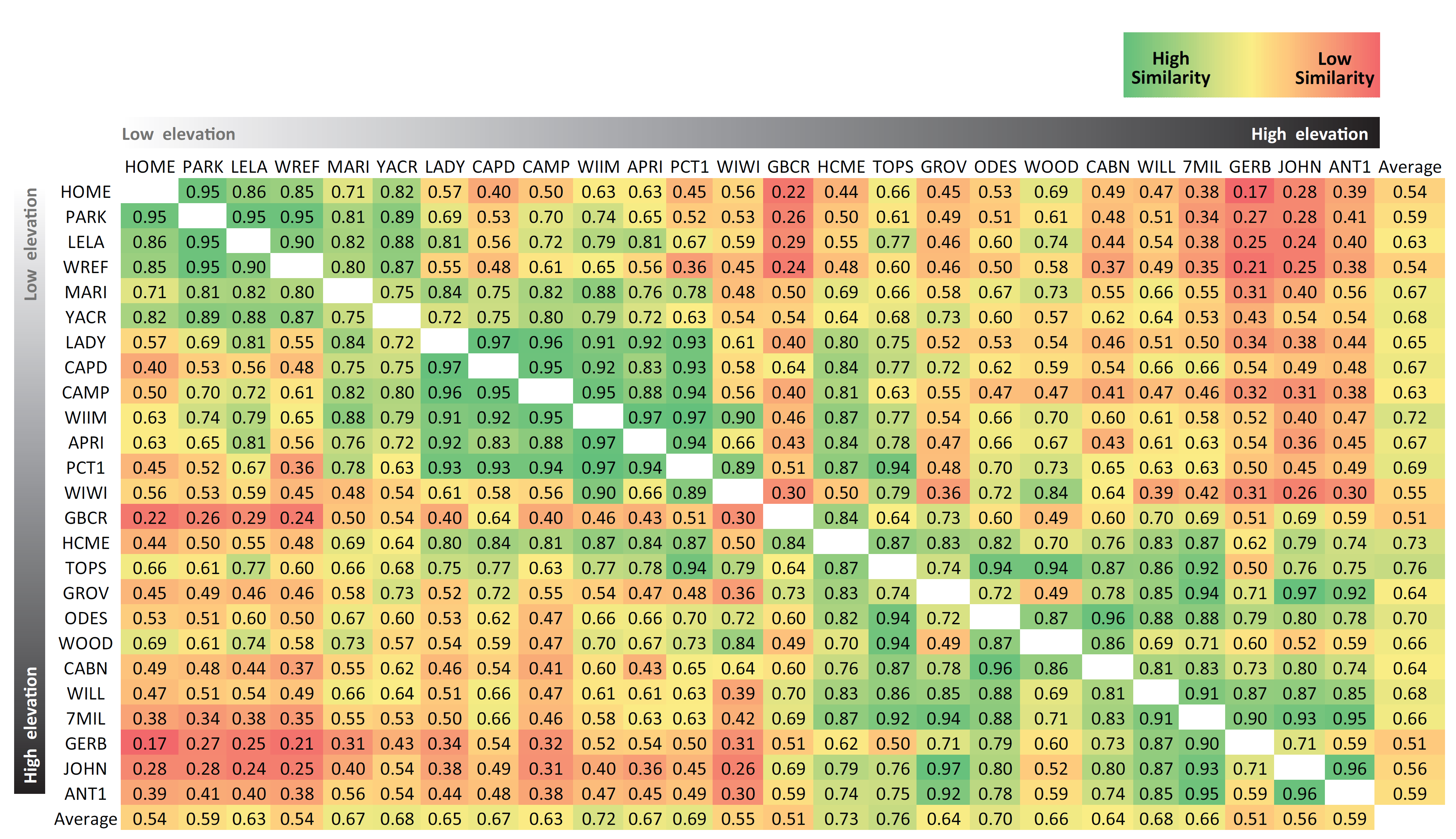


Figure S2. Results from the Chao’s abundance-based Jaccard community similarity analysis assessing molting bird communities at the 25 capture stations (z-transformed values and arranged by elevation). Lower (-) and higher (+) values represent dissimilar and similar bird communities, respectively.


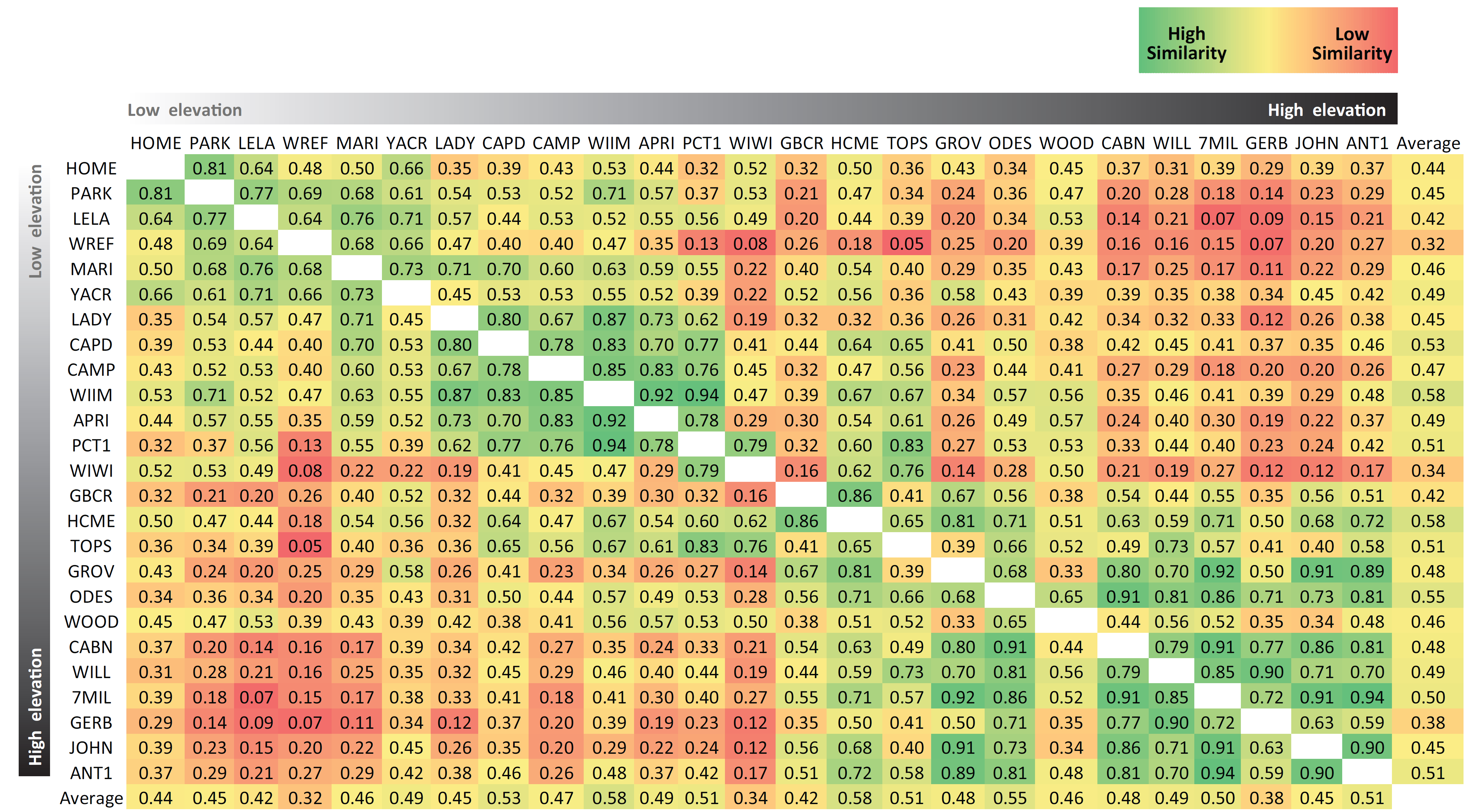


Figure S3. Results from the Chao’s abundance-based Jaccard community similarity analysis assessing naïve bird communities at the 25 capture stations (z-transformed values and arranged by elevation). Lower (-) and higher (+) values represent dissimilar and similar bird communities, respectively.


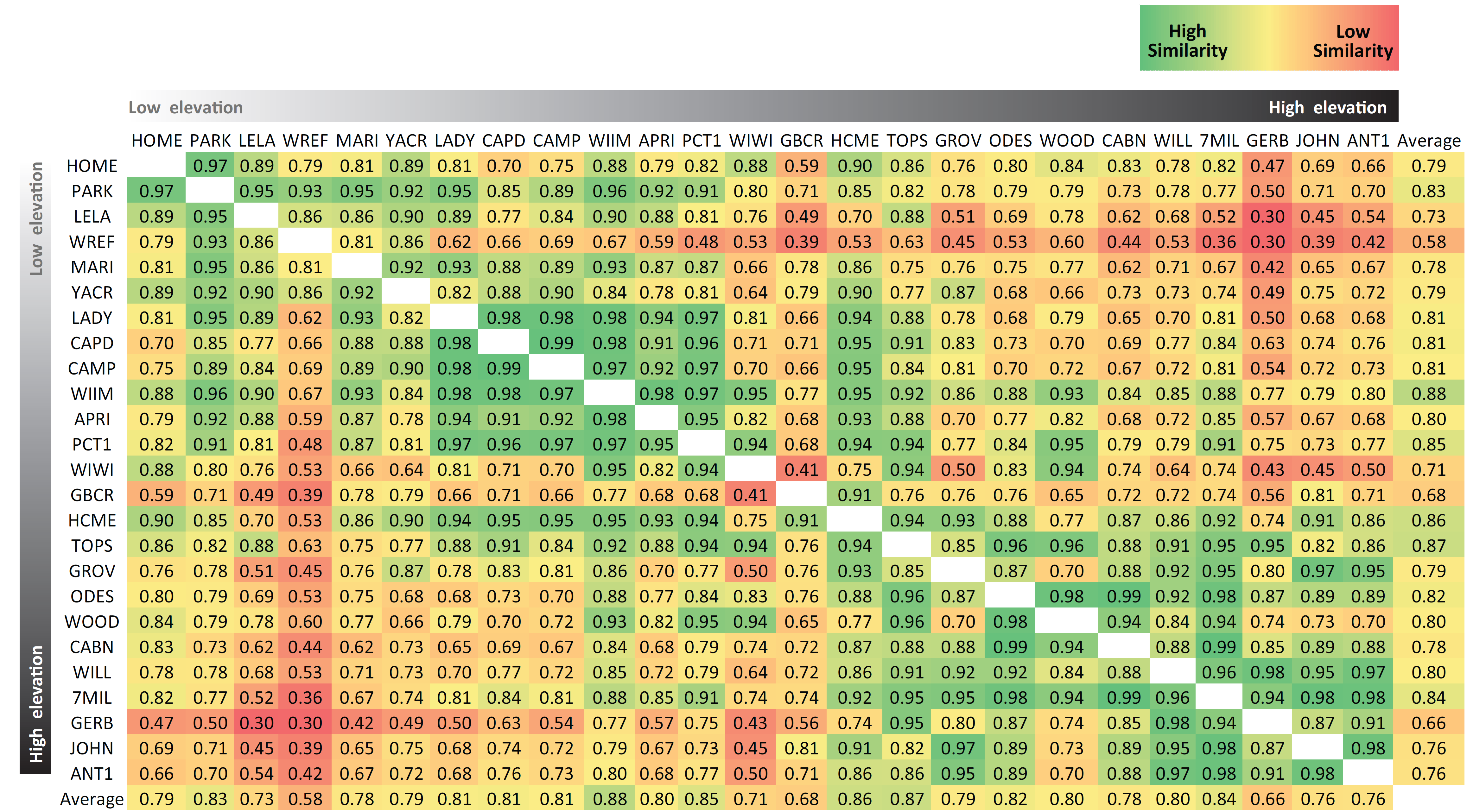


Figure S4. Detrended Correspondence Analysis (DCA) ordination for breeding bird communities denoted with convex hull polygons encompassing regions with more than five banding stations. Data used in the DCA came from captured individuals from May-October.

Figure S5. Detrended Correspondence Analysis (DCA) ordination for molting bird communities denoted with convex hull polygons encompassing regions with more than five banding stations. Data used in the DCA came from captured individuals from May-October.

Figure S6. Detrended Correspondence Analysis (DCA) ordination for naïve bird communities denoted with convex hull polygons encompassing regions with more than five banding stations. Data used in the DCA came from captured individuals from May-October.

Table S1. List of species included in the naïve (total) bird community and their respective percent of the total captures (standardized by birds per year of effort) for each of the 25 stations from May through October.

Table S1 continued. List of species included in the naïve (total) bird community and their respective percent of the total captures (standardized by birds per year of effort) for each of the 25 stations in southern Oregon and northern California.

Table S1 continued. continued. List of species included in the naïve (total) bird community and their respective percent of the total captures (standardized by birds per year of effort) for each of the 25 stations in southern Oregon and northern California.

Table S2. List of breeding bird species and their respective percent of the total captures (standardized by birds per year of effort) for each of the 25 stations in southern Oregon and northern California.

Table S2 continued. List of breeding bird species and their respective percent of the total captures (standardized by birds per year of effort) for each of the 25 stations in southern Oregon and northern California.

Table S2 continued. List of breeding bird species and their respective percent of the total captures (standardized by birds per year of effort) for each of the 25 stations in southern Oregon and northern California.

Table S3. List of molting bird species and their respective percent of the total captures (standardized by birds per year of effort) for each of the 25 stations southern Oregon and northern California.

Table S3 continued. List of molting bird species and their respective percent of the total captures (standardized by birds per year of effort) for each of the 25 stations southern Oregon and northern California.

Table S3 continued. List of molting bird species and their respective percent of the total captures (standardized by birds per year of effort) for each of the 25 stations southern Oregon and northern California.

Table S4. Species diversity statistics for the 25 capture stations in northern California and Southern Oregon.

|  | Naïve | | | Breeding | | | Molting | | |
| --- | --- | --- | --- | --- | --- | --- | --- | --- | --- |
| Station | Rarefaction (extrapolated 40 years) | Chao I (10-year) | Shannon (10-year ) | Rarefaction (extrapolated 40 years) | Chao I (10-year) | Shannon (10-year) | Rarefaction (extrapolated 40 years) | Chao I (10-year) | Shannon (10-year ) |
| 7MIL | 71.44 | 66.55 | 3.28 | 55.27 | 50.27 | 2.82 | 43.93 | 40.62 | 2.50 |
| ANT1 | 64.29 | 56.31 | 3.14 | 50.13 | 47.45 | 2.75 | 39.02 | 40.09 | 2.74 |
| APRI | 52.80 | 54.09 | 3.15 | 35.71 | 32.41 | 2.64 | 27.07 | 23.69 | 2.76 |
| CABN | 80.35 | 72.62 | 3.41 | 59.20 | 54.54 | 2.79 | 45.16 | 43.89 | 2.94 |
| CAMP | 57.97 | 59.85 | 3.05 | 42.88 | 33.86 | 2.41 | 46.69 | 48.15 | 2.67 |
| CAPD | 63.78 | 57.35 | 3.17 | 47.99 | 43.10 | 2.61 | 29.60 | 26.50 | 2.73 |
| GBCR | 48.56 | 51.41 | 2.66 | 25.36 | 24.92 | 2.38 | 24.64 | 21.46 | 2.48 |
| GERB | 57.71 | 56.85 | 3.19 | 47.04 | 49.58 | 2.79 | 33.33 | 33.8 | 2.51 |
| GROV | 69.81 | 65.12 | 3.08 | 53.63 | 38.75 | 2.88 | 37.56 | 27.97 | 2.16 |
| HCME | 69.36 | 60.53 | 3.08 | 44.85 | 41.55 | 2.82 | 44.36 | 32.68 | 2.52 |
| HOME | 69.43 | 67.39 | 3.17 | 57.11 | 54.17 | 2.58 | 40.64 | 34.19 | 2.83 |
| JOHN | 60.27 | 62.99 | 3.12 | 46.23 | 41.98 | 2.72 | 46.23 | 41.98 | 2.72 |
| LADY | 53.68 | 55.28 | 2.94 | 41.11 | 34.56 | 2.41 | 33.17 | 35.84 | 2.53 |
| LELA | 59.04 | 51.00 | 2.48 | 30.45 | 31.00 | 2.13 | 19.79 | 20.30 | 2.33 |
| MARI | 43.67 | 41.12 | 2.42 | 30.55 | 28.06 | 2.14 | 15.83 | 16.12 | 2.06 |
| ODES | 74.90 | 71.99 | 3.36 | 54.67 | 53.69 | 2.85 | 50.46 | 45.58 | 3.08 |
| PARK | 59.11 | 53.11 | 2.66 | 34.76 | 30.01 | 2.20 | 34.11 | 29.08 | 2.09 |
| PCT1 | 76.93 | 68.95 | 3.16 | 48.56 | 44.29 | 2.61 | 36.76 | 36.06 | 2.80 |
| TOPS | 78.15 | 76.00 | 2.95 | 69.34 | 58.12 | 2.50 | 53.48 | 51.75 | 2.34 |
| WIIM | 70.45 | 64.96 | 3.03 | 48.12 | 49.12 | 2.47 | 35.29 | 36.09 | 2.62 |
| WILL | 64.29 | 65.2 | 3.24 | 53.32 | 49.12 | 2.75 | 41.06 | 35.41 | 2.79 |
| WIWI | 82.52 | 75.96 | 3.25 | 45.18 | 43.31 | 2.51 | 41.71 | 41.57 | 2.31 |
| WOOD | 67.5 | 67.06 | 2.74 | 45.44 | 45.19 | 1.98 | 35.23 | 30.57 | 2.56 |
| WREF | 43.36 | 36.53 | 2.24 | 29.09 | 26.31 | 2.03 | 16.36 | 11.18 | 1.98 |
| YACR | 56.98 | 59.5 | 2.73 | 42.14 | 42.75 | 2.47 | 32.56 | 27.95 | 2.57 |

Table S5. Detrended Correspondence Analysis (DCA) eigenvalues and axis lengths.

|  | *Naïve Community* | | | |
| --- | --- | --- | --- | --- |
|  | DCA1 | DCA2 | DCA3 | DCA4 |
| Eigenvalues | 0.33 | 0.26 | 0.15 | 0.12 |
| Axis lengths | 3.57 | 3.70 | 4.28 | 3.95 |
|  |  |  |  |  |
|  | *Breeding Community* | | | |
|  | DCA1 | DCA2 | DCA3 | DCA4 |
| Eigenvalues | 0.41 | 0.33 | 0.23 | 0.14 |
| Axis lengths | 4.99 | 3.74 | 4.32 | 4.07 |
|  |  |  |  |  |
|  | *Molting Community* | | | |
|  | DCA1 | DCA2 | DCA3 | DCA4 |
| Eigenvalues | 0.62 | 0.32 | 0.21 | 0.16 |
| Axis lengths | 4.41 | 4.16 | 2.98 | 3.63 |

Table S6. Stations and their direction cosines of the vectors used in the ordination, squared correlation coeﬃcient (r^2^), and respective p-values for birds captured between May through October.
