# Supplementary material for: A novel approach to understanding bird communities using informed diversity estimates at local and regional scales in northern California and southern Oregon
Source: Ecol Evol. 2019 Mar 15;9(8):4431–42. doi: 10.1002/ece3.5008 (PMC6476868; doi:10.1002/ece3.5008)
Supplement: Supplementary file 2 [file ECE3-9-4431-s002.docx]

Appendix 1. List of study species captured at 25 capture stations in southern Oregon and northern California.

Appendix 1, Table 1. List of species included in the naïve (total) bird community and their respective percent of the total captures (standardized by birds per year of effort) for each of the 25 stations from May through October.

| Species | 7MIL | ANT1 | APRI | CABN | CAMP | CAPD | GBCR | GERB | GROV | HCME | HOME | JOHN | LADY | LELA | MARI | ODES | PARK | PCT1 | TOPS | WIIM | WILL | WIWI | WOOD | WREF | YACR |
| --- | --- | --- | --- | --- | --- | --- | --- | --- | --- | --- | --- | --- | --- | --- | --- | --- | --- | --- | --- | --- | --- | --- | --- | --- | --- |
| Acorn Woodpecker | 0.000 | 0.000 | 0.000 | 0.000 | 0.000 | 0.094 | 0.000 | 0.000 | 0.000 | 0.000 | 0.000 | 0.000 | 0.000 | 0.000 | 0.000 | 0.000 | 0.000 | 0.000 | 0.201 | 0.000 | 0.000 | 0.705 | 0.000 | 0.000 | 0.000 |
| Alder/Willow Flycatcher(Traill's) | 0.000 | 0.000 | 0.000 | 0.000 | 0.040 | 0.037 | 0.000 | 0.000 | 0.000 | 0.000 | 0.000 | 0.000 | 0.085 | 0.000 | 0.000 | 0.000 | 0.000 | 0.839 | 0.000 | 0.000 | 0.000 | 0.000 | 0.000 | 0.000 | 0.000 |
| Allen's Hummingbird | 0.010 | 0.016 | 0.000 | 0.000 | 0.072 | 0.082 | 0.000 | 0.000 | 0.034 | 0.005 | 0.494 | 0.004 | 0.082 | 0.031 | 0.021 | 0.000 | 0.059 | 0.025 | 0.007 | 0.008 | 0.009 | 0.000 | 0.000 | 0.004 | 0.037 |
| American Goldfinch | 0.002 | 0.000 | 0.003 | 0.000 | 0.000 | 0.000 | 0.000 | 0.000 | 0.000 | 0.000 | 0.236 | 0.000 | 0.001 | 0.031 | 0.004 | 0.000 | 0.041 | 0.002 | 0.003 | 0.002 | 0.002 | 0.585 | 0.084 | 0.002 | 0.001 |
| American Robin | 0.066 | 0.024 | 0.041 | 0.058 | 0.020 | 0.011 | 0.004 | 0.046 | 0.053 | 0.014 | 0.062 | 0.012 | 0.009 | 0.025 | 0.020 | 0.108 | 0.034 | 0.010 | 0.028 | 0.076 | 0.061 | 0.013 | 0.169 | 0.009 | 0.027 |
| Anna's Hummingbird | 0.000 | 0.000 | 0.007 | 0.000 | 0.033 | 0.014 | 0.000 | 0.000 | 0.013 | 0.000 | 0.065 | 0.005 | 0.006 | 0.021 | 0.017 | 0.000 | 0.027 | 0.000 | 0.005 | 0.024 | 0.000 | 0.752 | 0.000 | 0.004 | 0.007 |
| Ash-throated Flycatcher | 0.000 | 0.000 | 0.000 | 0.033 | 0.000 | 0.035 | 0.000 | 0.000 | 0.000 | 0.000 | 0.191 | 0.000 | 0.000 | 0.000 | 0.000 | 0.000 | 0.000 | 0.099 | 0.000 | 0.380 | 0.000 | 0.263 | 0.000 | 0.000 | 0.000 |
| Bewick's Wren | 0.000 | 0.003 | 0.027 | 0.007 | 0.000 | 0.000 | 0.000 | 0.000 | 0.000 | 0.020 | 0.035 | 0.000 | 0.000 | 0.094 | 0.000 | 0.004 | 0.000 | 0.238 | 0.004 | 0.016 | 0.000 | 0.439 | 0.008 | 0.000 | 0.105 |
| Black Phoebe | 0.000 | 0.000 | 0.000 | 0.000 | 0.013 | 0.028 | 0.000 | 0.000 | 0.000 | 0.000 | 0.568 | 0.000 | 0.022 | 0.014 | 0.000 | 0.000 | 0.079 | 0.070 | 0.000 | 0.016 | 0.000 | 0.070 | 0.000 | 0.009 | 0.112 |
| Black-capped Chickadee | 0.002 | 0.000 | 0.096 | 0.039 | 0.040 | 0.002 | 0.016 | 0.000 | 0.000 | 0.019 | 0.020 | 0.000 | 0.005 | 0.000 | 0.026 | 0.059 | 0.010 | 0.067 | 0.000 | 0.137 | 0.000 | 0.380 | 0.081 | 0.000 | 0.000 |
| Black-headed Grosbeak | 0.046 | 0.002 | 0.051 | 0.060 | 0.036 | 0.066 | 0.033 | 0.046 | 0.024 | 0.040 | 0.008 | 0.014 | 0.021 | 0.044 | 0.017 | 0.074 | 0.001 | 0.039 | 0.091 | 0.064 | 0.050 | 0.094 | 0.058 | 0.009 | 0.014 |
| Black-throated Gray Warbler | 0.000 | 0.000 | 0.000 | 0.018 | 0.000 | 0.019 | 0.341 | 0.000 | 0.000 | 0.169 | 0.007 | 0.010 | 0.000 | 0.101 | 0.022 | 0.000 | 0.000 | 0.163 | 0.021 | 0.072 | 0.000 | 0.000 | 0.000 | 0.000 | 0.058 |
| Brewer's Blackbird | 0.064 | 0.000 | 0.000 | 0.015 | 0.000 | 0.000 | 0.000 | 0.022 | 0.000 | 0.000 | 0.130 | 0.000 | 0.000 | 0.000 | 0.000 | 0.000 | 0.000 | 0.015 | 0.000 | 0.000 | 0.000 | 0.715 | 0.016 | 0.000 | 0.024 |
| Brewer's Sparrow | 0.101 | 0.013 | 0.000 | 0.448 | 0.000 | 0.000 | 0.000 | 0.039 | 0.000 | 0.012 | 0.000 | 0.014 | 0.000 | 0.000 | 0.000 | 0.081 | 0.000 | 0.027 | 0.000 | 0.000 | 0.047 | 0.217 | 0.000 | 0.000 | 0.000 |
| Brown Creeper | 0.102 | 0.020 | 0.114 | 0.078 | 0.000 | 0.000 | 0.000 | 0.145 | 0.007 | 0.000 | 0.000 | 0.057 | 0.000 | 0.000 | 0.000 | 0.174 | 0.011 | 0.004 | 0.049 | 0.016 | 0.053 | 0.000 | 0.008 | 0.060 | 0.102 |
| Brown-headed Cowbird | 0.013 | 0.000 | 0.027 | 0.041 | 0.002 | 0.000 | 0.000 | 0.006 | 0.000 | 0.000 | 0.142 | 0.001 | 0.006 | 0.052 | 0.004 | 0.062 | 0.048 | 0.014 | 0.012 | 0.018 | 0.026 | 0.158 | 0.351 | 0.005 | 0.012 |
| Bullock's Oriole | 0.003 | 0.000 | 0.041 | 0.014 | 0.000 | 0.003 | 0.000 | 0.000 | 0.000 | 0.001 | 0.127 | 0.000 | 0.004 | 0.034 | 0.000 | 0.029 | 0.010 | 0.009 | 0.126 | 0.014 | 0.000 | 0.550 | 0.035 | 0.000 | 0.000 |
| Bushtit | 0.000 | 0.000 | 0.028 | 0.010 | 0.002 | 0.009 | 0.028 | 0.044 | 0.001 | 0.063 | 0.022 | 0.000 | 0.002 | 0.020 | 0.003 | 0.018 | 0.001 | 0.062 | 0.104 | 0.025 | 0.028 | 0.465 | 0.001 | 0.000 | 0.062 |
| California Quail | 0.000 | 0.000 | 0.000 | 0.000 | 0.095 | 0.069 | 0.000 | 0.000 | 0.000 | 0.000 | 0.236 | 0.000 | 0.008 | 0.000 | 0.000 | 0.000 | 0.019 | 0.013 | 0.000 | 0.012 | 0.000 | 0.468 | 0.007 | 0.000 | 0.073 |
| California Towhee | 0.000 | 0.000 | 0.000 | 0.000 | 0.000 | 0.000 | 0.000 | 0.000 | 0.000 | 0.000 | 0.000 | 0.000 | 0.000 | 0.059 | 0.000 | 0.000 | 0.000 | 0.000 | 0.000 | 0.000 | 0.000 | 0.882 | 0.000 | 0.000 | 0.059 |
| Calliope Hummingbird | 0.000 | 0.034 | 0.000 | 0.000 | 0.000 | 0.000 | 0.000 | 0.208 | 0.000 | 0.000 | 0.009 | 0.000 | 0.000 | 0.019 | 0.000 | 0.012 | 0.000 | 0.000 | 0.463 | 0.011 | 0.150 | 0.095 | 0.000 | 0.000 | 0.000 |
| Cassin's Finch | 0.049 | 0.128 | 0.000 | 0.036 | 0.000 | 0.000 | 0.000 | 0.079 | 0.000 | 0.008 | 0.000 | 0.000 | 0.000 | 0.000 | 0.000 | 0.055 | 0.000 | 0.000 | 0.333 | 0.000 | 0.166 | 0.146 | 0.000 | 0.000 | 0.000 |
| Cassin's Vireo | 0.019 | 0.064 | 0.002 | 0.091 | 0.048 | 0.012 | 0.004 | 0.032 | 0.054 | 0.066 | 0.002 | 0.021 | 0.010 | 0.011 | 0.002 | 0.041 | 0.000 | 0.012 | 0.342 | 0.025 | 0.090 | 0.033 | 0.003 | 0.000 | 0.015 |
| Cedar Waxwing | 0.000 | 0.000 | 0.010 | 0.009 | 0.000 | 0.000 | 0.000 | 0.000 | 0.002 | 0.004 | 0.227 | 0.000 | 0.014 | 0.055 | 0.250 | 0.055 | 0.043 | 0.030 | 0.003 | 0.136 | 0.005 | 0.000 | 0.041 | 0.011 | 0.106 |
| Chestnut-backed Chickadee | 0.025 | 0.000 | 0.000 | 0.075 | 0.005 | 0.000 | 0.022 | 0.000 | 0.088 | 0.022 | 0.329 | 0.027 | 0.002 | 0.096 | 0.049 | 0.000 | 0.080 | 0.000 | 0.000 | 0.000 | 0.000 | 0.000 | 0.000 | 0.022 | 0.160 |
| Chipping Sparrow | 0.086 | 0.013 | 0.000 | 0.076 | 0.007 | 0.005 | 0.000 | 0.324 | 0.019 | 0.000 | 0.007 | 0.010 | 0.000 | 0.000 | 0.000 | 0.005 | 0.000 | 0.005 | 0.125 | 0.004 | 0.238 | 0.076 | 0.000 | 0.000 | 0.000 |
| Common Yellowthroat | 0.002 | 0.000 | 0.020 | 0.058 | 0.004 | 0.010 | 0.000 | 0.000 | 0.002 | 0.001 | 0.010 | 0.000 | 0.004 | 0.000 | 0.008 | 0.058 | 0.002 | 0.034 | 0.007 | 0.031 | 0.005 | 0.647 | 0.098 | 0.002 | 0.000 |
| Dark-eyed Junco | 0.133 | 0.088 | 0.004 | 0.115 | 0.003 | 0.002 | 0.056 | 0.099 | 0.176 | 0.032 | 0.021 | 0.159 | 0.002 | 0.000 | 0.000 | 0.037 | 0.002 | 0.003 | 0.004 | 0.002 | 0.041 | 0.000 | 0.004 | 0.000 | 0.019 |
| Downy Woodpecker | 0.022 | 0.009 | 0.048 | 0.033 | 0.020 | 0.016 | 0.002 | 0.010 | 0.008 | 0.007 | 0.039 | 0.000 | 0.010 | 0.031 | 0.022 | 0.017 | 0.028 | 0.031 | 0.047 | 0.064 | 0.023 | 0.398 | 0.078 | 0.010 | 0.029 |
| Dusky Flycatcher | 0.047 | 0.007 | 0.000 | 0.210 | 0.000 | 0.019 | 0.000 | 0.031 | 0.317 | 0.001 | 0.002 | 0.024 | 0.007 | 0.000 | 0.000 | 0.068 | 0.000 | 0.024 | 0.041 | 0.004 | 0.016 | 0.133 | 0.047 | 0.000 | 0.002 |
| European Starling | 0.010 | 0.000 | 0.000 | 0.000 | 0.000 | 0.000 | 0.000 | 0.000 | 0.000 | 0.000 | 0.281 | 0.000 | 0.000 | 0.000 | 0.000 | 0.009 | 0.075 | 0.000 | 0.011 | 0.000 | 0.000 | 0.603 | 0.010 | 0.000 | 0.000 |
| Evening Grosbeak | 0.062 | 0.385 | 0.000 | 0.007 | 0.000 | 0.000 | 0.000 | 0.000 | 0.007 | 0.000 | 0.000 | 0.366 | 0.000 | 0.000 | 0.000 | 0.022 | 0.000 | 0.000 | 0.000 | 0.000 | 0.084 | 0.058 | 0.008 | 0.000 | 0.000 |
| Fox Sparrow | 0.012 | 0.021 | 0.032 | 0.030 | 0.115 | 0.038 | 0.000 | 0.001 | 0.025 | 0.011 | 0.103 | 0.034 | 0.048 | 0.009 | 0.027 | 0.022 | 0.093 | 0.038 | 0.007 | 0.194 | 0.018 | 0.102 | 0.005 | 0.007 | 0.007 |
| Golden-crowned Kinglet | 0.113 | 0.031 | 0.000 | 0.217 | 0.006 | 0.000 | 0.006 | 0.036 | 0.114 | 0.015 | 0.119 | 0.222 | 0.000 | 0.000 | 0.000 | 0.025 | 0.020 | 0.000 | 0.000 | 0.004 | 0.000 | 0.000 | 0.000 | 0.053 | 0.020 |
| Golden-crowned Sparrow | 0.016 | 0.017 | 0.014 | 0.020 | 0.056 | 0.042 | 0.000 | 0.003 | 0.027 | 0.004 | 0.032 | 0.009 | 0.022 | 0.001 | 0.000 | 0.012 | 0.006 | 0.146 | 0.022 | 0.106 | 0.005 | 0.437 | 0.004 | 0.000 | 0.000 |
| Grasshopper Sparrow | 0.000 | 0.000 | 0.000 | 0.000 | 0.000 | 0.000 | 0.000 | 0.000 | 0.000 | 0.000 | 0.000 | 0.000 | 0.000 | 0.000 | 0.000 | 0.000 | 0.000 | 0.000 | 0.000 | 0.000 | 1.000 | 0.000 | 0.000 | 0.000 | 0.000 |
| Gray Flycatcher | 0.090 | 0.011 | 0.000 | 0.048 | 0.000 | 0.000 | 0.000 | 0.228 | 0.109 | 0.000 | 0.009 | 0.000 | 0.015 | 0.000 | 0.000 | 0.012 | 0.000 | 0.024 | 0.069 | 0.000 | 0.055 | 0.290 | 0.039 | 0.000 | 0.000 |
| Gray Jay | 0.040 | 0.036 | 0.000 | 0.113 | 0.000 | 0.000 | 0.000 | 0.000 | 0.000 | 0.000 | 0.000 | 0.725 | 0.000 | 0.000 | 0.000 | 0.000 | 0.000 | 0.000 | 0.000 | 0.000 | 0.086 | 0.000 | 0.000 | 0.000 | 0.000 |
| Green-tailed Towhee | 0.000 | 0.019 | 0.000 | 0.000 | 0.000 | 0.000 | 0.000 | 0.059 | 0.020 | 0.000 | 0.000 | 0.044 | 0.000 | 0.000 | 0.000 | 0.000 | 0.000 | 0.000 | 0.023 | 0.018 | 0.794 | 0.000 | 0.022 | 0.000 | 0.000 |
| Hairy Woodpecker | 0.101 | 0.019 | 0.059 | 0.054 | 0.000 | 0.014 | 0.039 | 0.118 | 0.014 | 0.018 | 0.049 | 0.014 | 0.000 | 0.054 | 0.108 | 0.054 | 0.034 | 0.041 | 0.015 | 0.018 | 0.039 | 0.000 | 0.029 | 0.020 | 0.087 |
| Hammond's Flycatcher | 0.090 | 0.047 | 0.000 | 0.111 | 0.004 | 0.003 | 0.004 | 0.025 | 0.189 | 0.005 | 0.000 | 0.189 | 0.000 | 0.000 | 0.000 | 0.061 | 0.000 | 0.073 | 0.020 | 0.003 | 0.057 | 0.116 | 0.003 | 0.000 | 0.000 |
| Hermit Thrush | 0.019 | 0.019 | 0.016 | 0.085 | 0.079 | 0.045 | 0.038 | 0.016 | 0.049 | 0.040 | 0.102 | 0.084 | 0.036 | 0.005 | 0.012 | 0.041 | 0.033 | 0.087 | 0.035 | 0.020 | 0.012 | 0.080 | 0.020 | 0.010 | 0.016 |
| Hermit Warbler | 0.082 | 0.123 | 0.000 | 0.253 | 0.000 | 0.000 | 0.043 | 0.000 | 0.168 | 0.075 | 0.000 | 0.193 | 0.000 | 0.000 | 0.000 | 0.036 | 0.000 | 0.000 | 0.000 | 0.000 | 0.002 | 0.000 | 0.000 | 0.000 | 0.026 |
| House Finch | 0.000 | 0.000 | 0.004 | 0.000 | 0.000 | 0.000 | 0.000 | 0.000 | 0.000 | 0.000 | 0.517 | 0.000 | 0.000 | 0.005 | 0.000 | 0.000 | 0.000 | 0.000 | 0.000 | 0.014 | 0.000 | 0.441 | 0.000 | 0.000 | 0.020 |
| House Sparrow | 0.000 | 0.000 | 0.000 | 0.000 | 0.000 | 0.000 | 0.000 | 0.000 | 0.000 | 0.000 | 0.036 | 0.000 | 0.000 | 0.000 | 0.000 | 0.000 | 0.000 | 0.000 | 0.000 | 0.006 | 0.000 | 0.958 | 0.000 | 0.000 | 0.000 |
| House Wren | 0.442 | 0.019 | 0.104 | 0.005 | 0.000 | 0.000 | 0.000 | 0.015 | 0.000 | 0.114 | 0.000 | 0.005 | 0.000 | 0.000 | 0.000 | 0.143 | 0.000 | 0.026 | 0.064 | 0.000 | 0.023 | 0.000 | 0.038 | 0.000 | 0.000 |
| Hutton's Vireo | 0.000 | 0.017 | 0.000 | 0.009 | 0.013 | 0.029 | 0.065 | 0.000 | 0.018 | 0.096 | 0.222 | 0.000 | 0.000 | 0.000 | 0.000 | 0.000 | 0.018 | 0.009 | 0.010 | 0.000 | 0.000 | 0.000 | 0.000 | 0.090 | 0.403 |
| Lazuli Bunting | 0.156 | 0.075 | 0.033 | 0.017 | 0.008 | 0.007 | 0.000 | 0.007 | 0.072 | 0.110 | 0.006 | 0.100 | 0.041 | 0.002 | 0.000 | 0.010 | 0.000 | 0.023 | 0.193 | 0.014 | 0.013 | 0.091 | 0.004 | 0.006 | 0.011 |
| Lesser Goldfinch | 0.001 | 0.000 | 0.026 | 0.000 | 0.004 | 0.005 | 0.000 | 0.001 | 0.000 | 0.008 | 0.001 | 0.000 | 0.001 | 0.011 | 0.000 | 0.001 | 0.001 | 0.058 | 0.173 | 0.004 | 0.000 | 0.701 | 0.002 | 0.003 | 0.001 |
| Lewis's Woodpecker | 0.000 | 0.000 | 0.000 | 0.000 | 0.000 | 0.000 | 0.000 | 0.000 | 0.000 | 0.000 | 0.000 | 0.000 | 0.000 | 0.000 | 0.000 | 0.000 | 0.000 | 0.000 | 0.000 | 0.000 | 0.000 | 1.000 | 0.000 | 0.000 | 0.000 |
| Lincoln's Sparrow | 0.057 | 0.119 | 0.003 | 0.034 | 0.005 | 0.001 | 0.000 | 0.008 | 0.076 | 0.000 | 0.012 | 0.020 | 0.002 | 0.001 | 0.003 | 0.007 | 0.002 | 0.008 | 0.034 | 0.013 | 0.035 | 0.543 | 0.017 | 0.000 | 0.000 |
| MacGillivray's Warbler | 0.143 | 0.047 | 0.011 | 0.017 | 0.010 | 0.045 | 0.064 | 0.003 | 0.059 | 0.112 | 0.001 | 0.078 | 0.034 | 0.000 | 0.069 | 0.031 | 0.000 | 0.119 | 0.022 | 0.048 | 0.042 | 0.034 | 0.010 | 0.000 | 0.000 |
| Marsh Wren | 0.000 | 0.000 | 0.000 | 0.176 | 0.000 | 0.000 | 0.000 | 0.000 | 0.000 | 0.000 | 0.000 | 0.000 | 0.000 | 0.000 | 0.000 | 0.176 | 0.000 | 0.000 | 0.000 | 0.031 | 0.000 | 0.281 | 0.300 | 0.035 | 0.000 |
| Mountain Chickadee | 0.058 | 0.060 | 0.000 | 0.102 | 0.000 | 0.000 | 0.000 | 0.438 | 0.000 | 0.000 | 0.000 | 0.047 | 0.000 | 0.000 | 0.000 | 0.075 | 0.000 | 0.002 | 0.007 | 0.003 | 0.183 | 0.000 | 0.024 | 0.000 | 0.000 |
| Mountain Quail | 0.000 | 0.000 | 0.000 | 0.000 | 0.000 | 0.000 | 0.000 | 0.000 | 0.000 | 0.000 | 0.000 | 0.000 | 0.000 | 0.000 | 0.000 | 0.000 | 0.000 | 1.000 | 0.000 | 0.000 | 0.000 | 0.000 | 0.000 | 0.000 | 0.000 |
| Mourning Dove | 0.000 | 0.000 | 0.111 | 0.000 | 0.000 | 0.000 | 0.000 | 0.000 | 0.000 | 0.000 | 0.387 | 0.000 | 0.000 | 0.000 | 0.000 | 0.000 | 0.076 | 0.000 | 0.000 | 0.000 | 0.000 | 0.304 | 0.122 | 0.000 | 0.000 |
| Nashville Warbler | 0.233 | 0.080 | 0.001 | 0.102 | 0.003 | 0.010 | 0.105 | 0.020 | 0.036 | 0.118 | 0.003 | 0.099 | 0.002 | 0.000 | 0.000 | 0.046 | 0.000 | 0.035 | 0.034 | 0.002 | 0.033 | 0.030 | 0.006 | 0.000 | 0.000 |
| Northern Flicker | 0.023 | 0.036 | 0.031 | 0.089 | 0.043 | 0.018 | 0.000 | 0.068 | 0.025 | 0.004 | 0.178 | 0.032 | 0.000 | 0.000 | 0.005 | 0.038 | 0.004 | 0.046 | 0.024 | 0.026 | 0.019 | 0.237 | 0.041 | 0.000 | 0.014 |
| Oak Titmouse | 0.000 | 0.000 | 0.000 | 0.000 | 0.000 | 0.000 | 0.000 | 0.000 | 0.000 | 0.000 | 0.000 | 0.000 | 0.000 | 0.000 | 0.000 | 0.000 | 0.000 | 0.000 | 1.000 | 0.000 | 0.000 | 0.000 | 0.000 | 0.000 | 0.000 |
| Olive-sided Flycatcher | 0.000 | 0.021 | 0.000 | 0.068 | 0.000 | 0.024 | 0.000 | 0.000 | 0.045 | 0.020 | 0.397 | 0.000 | 0.028 | 0.000 | 0.000 | 0.114 | 0.000 | 0.023 | 0.000 | 0.000 | 0.078 | 0.182 | 0.000 | 0.000 | 0.000 |
| Orange-crowned Warbler | 0.080 | 0.082 | 0.013 | 0.094 | 0.019 | 0.011 | 0.001 | 0.036 | 0.020 | 0.032 | 0.056 | 0.060 | 0.016 | 0.007 | 0.011 | 0.027 | 0.015 | 0.019 | 0.018 | 0.076 | 0.077 | 0.118 | 0.056 | 0.023 | 0.035 |
| Pacific-slope/Cordilleran Flycatcher | 0.019 | 0.004 | 0.028 | 0.013 | 0.032 | 0.008 | 0.023 | 0.012 | 0.024 | 0.034 | 0.132 | 0.010 | 0.036 | 0.040 | 0.084 | 0.006 | 0.074 | 0.135 | 0.003 | 0.040 | 0.001 | 0.045 | 0.002 | 0.115 | 0.080 |
| Pacific Wren | 0.009 | 0.000 | 0.057 | 0.013 | 0.000 | 0.009 | 0.006 | 0.000 | 0.022 | 0.012 | 0.173 | 0.005 | 0.016 | 0.035 | 0.016 | 0.009 | 0.112 | 0.009 | 0.020 | 0.019 | 0.010 | 0.104 | 0.000 | 0.346 | 0.000 |
| Pileated Woodpecker | 0.000 | 0.000 | 0.000 | 0.000 | 0.000 | 0.000 | 0.577 | 0.000 | 0.000 | 0.000 | 0.000 | 0.423 | 0.000 | 0.000 | 0.000 | 0.000 | 0.000 | 0.000 | 0.000 | 0.000 | 0.000 | 0.000 | 0.000 | 0.000 | 0.000 |
| Pine Siskin | 0.026 | 0.037 | 0.000 | 0.048 | 0.002 | 0.000 | 0.000 | 0.000 | 0.078 | 0.030 | 0.341 | 0.026 | 0.000 | 0.000 | 0.000 | 0.013 | 0.034 | 0.001 | 0.029 | 0.000 | 0.002 | 0.329 | 0.003 | 0.000 | 0.000 |
| Purple Finch | 0.001 | 0.008 | 0.003 | 0.012 | 0.012 | 0.009 | 0.002 | 0.001 | 0.028 | 0.091 | 0.203 | 0.006 | 0.002 | 0.005 | 0.010 | 0.036 | 0.023 | 0.038 | 0.351 | 0.023 | 0.033 | 0.091 | 0.007 | 0.003 | 0.002 |
| Pygmy Nuthatch | 0.000 | 0.000 | 0.000 | 0.000 | 0.000 | 0.000 | 0.000 | 0.380 | 0.000 | 0.000 | 0.000 | 0.000 | 0.000 | 0.000 | 0.000 | 0.261 | 0.000 | 0.000 | 0.000 | 0.000 | 0.149 | 0.000 | 0.209 | 0.000 | 0.000 |
| Red Crossbill | 0.000 | 0.000 | 0.000 | 0.089 | 0.000 | 0.000 | 0.000 | 0.000 | 0.089 | 0.026 | 0.647 | 0.000 | 0.000 | 0.000 | 0.000 | 0.000 | 0.148 | 0.000 | 0.000 | 0.000 | 0.000 | 0.000 | 0.000 | 0.000 | 0.000 |
| Red-breasted Nuthatch | 0.072 | 0.068 | 0.000 | 0.347 | 0.000 | 0.000 | 0.004 | 0.121 | 0.041 | 0.002 | 0.015 | 0.155 | 0.000 | 0.000 | 0.000 | 0.065 | 0.000 | 0.000 | 0.006 | 0.000 | 0.101 | 0.000 | 0.003 | 0.000 | 0.000 |
| Red-breasted Sapsucker | 0.156 | 0.088 | 0.014 | 0.089 | 0.014 | 0.009 | 0.000 | 0.186 | 0.061 | 0.020 | 0.000 | 0.016 | 0.000 | 0.000 | 0.005 | 0.125 | 0.001 | 0.022 | 0.026 | 0.064 | 0.081 | 0.000 | 0.017 | 0.000 | 0.004 |
| Red-naped Sapsucker | 0.478 | 0.000 | 0.000 | 0.000 | 0.000 | 0.000 | 0.000 | 0.163 | 0.000 | 0.000 | 0.000 | 0.000 | 0.000 | 0.000 | 0.000 | 0.112 | 0.000 | 0.000 | 0.128 | 0.000 | 0.000 | 0.000 | 0.119 | 0.000 | 0.000 |
| Red-winged Blackbird | 0.001 | 0.000 | 0.000 | 0.121 | 0.000 | 0.000 | 0.000 | 0.000 | 0.000 | 0.000 | 0.001 | 0.000 | 0.000 | 0.000 | 0.000 | 0.049 | 0.000 | 0.001 | 0.001 | 0.000 | 0.000 | 0.687 | 0.140 | 0.000 | 0.000 |
| Ruby-crowned Kinglet | 0.039 | 0.107 | 0.000 | 0.060 | 0.047 | 0.020 | 0.004 | 0.160 | 0.051 | 0.011 | 0.136 | 0.031 | 0.009 | 0.000 | 0.000 | 0.005 | 0.024 | 0.074 | 0.053 | 0.056 | 0.010 | 0.050 | 0.005 | 0.044 | 0.004 |
| Ruffed Grouse | 0.000 | 0.000 | 0.000 | 0.500 | 0.000 | 0.000 | 0.000 | 0.000 | 0.000 | 0.000 | 0.000 | 0.000 | 0.000 | 0.000 | 0.000 | 0.500 | 0.000 | 0.000 | 0.000 | 0.000 | 0.000 | 0.000 | 0.000 | 0.000 | 0.000 |
| Rufous Hummingbird | 0.128 | 0.030 | 0.069 | 0.014 | 0.017 | 0.017 | 0.082 | 0.019 | 0.014 | 0.122 | 0.033 | 0.039 | 0.016 | 0.005 | 0.005 | 0.010 | 0.003 | 0.101 | 0.028 | 0.081 | 0.015 | 0.150 | 0.000 | 0.000 | 0.002 |
| Savannah Sparrow | 0.000 | 0.013 | 0.000 | 0.000 | 0.020 | 0.030 | 0.000 | 0.000 | 0.028 | 0.000 | 0.031 | 0.000 | 0.000 | 0.000 | 0.000 | 0.000 | 0.000 | 0.014 | 0.048 | 0.000 | 0.032 | 0.784 | 0.000 | 0.000 | 0.000 |
| Song Sparrow | 0.017 | 0.009 | 0.041 | 0.046 | 0.012 | 0.012 | 0.002 | 0.005 | 0.037 | 0.015 | 0.056 | 0.008 | 0.019 | 0.038 | 0.038 | 0.039 | 0.052 | 0.035 | 0.030 | 0.086 | 0.042 | 0.210 | 0.096 | 0.015 | 0.038 |
| Spotted Towhee | 0.009 | 0.003 | 0.071 | 0.020 | 0.065 | 0.073 | 0.023 | 0.041 | 0.002 | 0.049 | 0.019 | 0.002 | 0.049 | 0.001 | 0.006 | 0.036 | 0.012 | 0.107 | 0.055 | 0.161 | 0.033 | 0.156 | 0.004 | 0.003 | 0.003 |
| Steller's Jay | 0.026 | 0.038 | 0.000 | 0.092 | 0.012 | 0.115 | 0.099 | 0.041 | 0.084 | 0.050 | 0.000 | 0.030 | 0.005 | 0.006 | 0.044 | 0.052 | 0.000 | 0.000 | 0.087 | 0.011 | 0.073 | 0.000 | 0.030 | 0.048 | 0.058 |
| Swainson's Thrush | 0.002 | 0.001 | 0.022 | 0.003 | 0.054 | 0.022 | 0.031 | 0.000 | 0.008 | 0.011 | 0.122 | 0.008 | 0.040 | 0.131 | 0.130 | 0.016 | 0.095 | 0.032 | 0.003 | 0.058 | 0.001 | 0.027 | 0.005 | 0.089 | 0.089 |
| Townsend's Solitaire | 0.087 | 0.000 | 0.000 | 0.020 | 0.000 | 0.000 | 0.000 | 0.537 | 0.020 | 0.000 | 0.000 | 0.022 | 0.000 | 0.000 | 0.000 | 0.102 | 0.000 | 0.000 | 0.070 | 0.000 | 0.141 | 0.000 | 0.000 | 0.000 | 0.000 |
| Townsend's Warbler | 0.196 | 0.063 | 0.000 | 0.200 | 0.000 | 0.000 | 0.000 | 0.024 | 0.017 | 0.015 | 0.061 | 0.107 | 0.000 | 0.000 | 0.000 | 0.133 | 0.000 | 0.000 | 0.038 | 0.000 | 0.095 | 0.000 | 0.036 | 0.017 | 0.000 |
| Varied Thrush | 0.008 | 0.000 | 0.078 | 0.015 | 0.100 | 0.049 | 0.000 | 0.022 | 0.114 | 0.014 | 0.089 | 0.057 | 0.159 | 0.000 | 0.000 | 0.000 | 0.069 | 0.030 | 0.009 | 0.122 | 0.017 | 0.000 | 0.041 | 0.008 | 0.000 |
| Vesper Sparrow | 0.595 | 0.000 | 0.000 | 0.000 | 0.000 | 0.000 | 0.000 | 0.405 | 0.000 | 0.000 | 0.000 | 0.000 | 0.000 | 0.000 | 0.000 | 0.000 | 0.000 | 0.000 | 0.000 | 0.000 | 0.000 | 0.000 | 0.000 | 0.000 | 0.000 |
| Warbling Vireo | 0.071 | 0.059 | 0.011 | 0.064 | 0.005 | 0.005 | 0.062 | 0.017 | 0.051 | 0.190 | 0.015 | 0.066 | 0.002 | 0.028 | 0.037 | 0.041 | 0.004 | 0.011 | 0.158 | 0.013 | 0.022 | 0.056 | 0.009 | 0.000 | 0.002 |
| Western Bluebird | 0.000 | 0.000 | 0.404 | 0.000 | 0.000 | 0.000 | 0.000 | 0.000 | 0.278 | 0.000 | 0.000 | 0.000 | 0.000 | 0.000 | 0.000 | 0.000 | 0.000 | 0.000 | 0.318 | 0.000 | 0.000 | 0.000 | 0.000 | 0.000 | 0.000 |
| Western Scrub-Jay | 0.000 | 0.000 | 0.080 | 0.000 | 0.133 | 0.125 | 0.000 | 0.000 | 0.000 | 0.003 | 0.000 | 0.000 | 0.018 | 0.000 | 0.000 | 0.000 | 0.000 | 0.069 | 0.021 | 0.029 | 0.000 | 0.497 | 0.008 | 0.000 | 0.018 |
| Western Tanager | 0.037 | 0.008 | 0.010 | 0.282 | 0.031 | 0.069 | 0.007 | 0.020 | 0.011 | 0.023 | 0.007 | 0.064 | 0.013 | 0.007 | 0.004 | 0.043 | 0.001 | 0.036 | 0.143 | 0.028 | 0.036 | 0.102 | 0.012 | 0.002 | 0.005 |
| Western Wood-Pewee | 0.073 | 0.019 | 0.079 | 0.145 | 0.005 | 0.001 | 0.002 | 0.009 | 0.006 | 0.010 | 0.020 | 0.002 | 0.005 | 0.026 | 0.002 | 0.100 | 0.009 | 0.019 | 0.084 | 0.010 | 0.049 | 0.186 | 0.123 | 0.000 | 0.017 |
| White-breasted Nuthatch | 0.000 | 0.000 | 0.000 | 0.000 | 0.000 | 0.000 | 0.000 | 0.114 | 0.000 | 0.000 | 0.000 | 0.000 | 0.000 | 0.000 | 0.000 | 0.052 | 0.000 | 0.000 | 0.357 | 0.000 | 0.268 | 0.209 | 0.000 | 0.000 | 0.000 |
| White-crowned Sparrow | 0.058 | 0.031 | 0.002 | 0.067 | 0.008 | 0.049 | 0.001 | 0.034 | 0.006 | 0.030 | 0.117 | 0.034 | 0.008 | 0.004 | 0.001 | 0.029 | 0.035 | 0.023 | 0.044 | 0.051 | 0.061 | 0.282 | 0.014 | 0.002 | 0.010 |
| White-headed Woodpecker | 0.000 | 0.133 | 0.000 | 0.706 | 0.000 | 0.000 | 0.000 | 0.000 | 0.000 | 0.000 | 0.000 | 0.000 | 0.000 | 0.000 | 0.000 | 0.000 | 0.000 | 0.000 | 0.161 | 0.000 | 0.000 | 0.000 | 0.000 | 0.000 | 0.000 |
| Williamson's Sapsucker | 0.000 | 0.000 | 0.000 | 0.375 | 0.000 | 0.000 | 0.000 | 0.000 | 0.000 | 0.000 | 0.000 | 0.000 | 0.000 | 0.000 | 0.000 | 0.625 | 0.000 | 0.000 | 0.000 | 0.000 | 0.000 | 0.000 | 0.000 | 0.000 | 0.000 |
| Willow Flycatcher | 0.058 | 0.002 | 0.016 | 0.092 | 0.022 | 0.017 | 0.001 | 0.012 | 0.004 | 0.019 | 0.002 | 0.007 | 0.018 | 0.004 | 0.000 | 0.024 | 0.001 | 0.040 | 0.055 | 0.094 | 0.010 | 0.418 | 0.083 | 0.000 | 0.001 |
| Wilson's Warbler | 0.027 | 0.075 | 0.011 | 0.030 | 0.005 | 0.012 | 0.003 | 0.007 | 0.029 | 0.015 | 0.031 | 0.029 | 0.016 | 0.024 | 0.042 | 0.054 | 0.015 | 0.069 | 0.050 | 0.014 | 0.013 | 0.282 | 0.050 | 0.053 | 0.041 |
| Wrentit | 0.000 | 0.000 | 0.071 | 0.000 | 0.123 | 0.055 | 0.000 | 0.000 | 0.000 | 0.014 | 0.153 | 0.000 | 0.084 | 0.048 | 0.067 | 0.000 | 0.083 | 0.007 | 0.000 | 0.117 | 0.000 | 0.080 | 0.000 | 0.038 | 0.059 |
| Yellow Warbler | 0.016 | 0.003 | 0.061 | 0.057 | 0.013 | 0.005 | 0.000 | 0.003 | 0.003 | 0.006 | 0.024 | 0.001 | 0.059 | 0.006 | 0.001 | 0.088 | 0.005 | 0.113 | 0.111 | 0.110 | 0.023 | 0.164 | 0.128 | 0.000 | 0.000 |
| Yellow-breasted Chat | 0.000 | 0.000 | 0.106 | 0.000 | 0.092 | 0.073 | 0.000 | 0.000 | 0.000 | 0.003 | 0.000 | 0.000 | 0.134 | 0.028 | 0.025 | 0.000 | 0.002 | 0.229 | 0.019 | 0.115 | 0.000 | 0.165 | 0.001 | 0.000 | 0.006 |
| Yellow-headed Blackbird | 0.000 | 0.000 | 0.000 | 0.000 | 0.000 | 0.000 | 0.000 | 0.000 | 0.000 | 0.000 | 0.000 | 0.000 | 0.000 | 0.000 | 0.000 | 0.000 | 0.000 | 0.000 | 0.000 | 0.000 | 0.000 | 0.000 | 1.000 | 0.000 | 0.000 |
| Yellow-rumped Warbler | 0.089 | 0.057 | 0.029 | 0.178 | 0.011 | 0.004 | 0.000 | 0.025 | 0.033 | 0.002 | 0.158 | 0.063 | 0.005 | 0.001 | 0.000 | 0.047 | 0.011 | 0.007 | 0.080 | 0.030 | 0.117 | 0.026 | 0.025 | 0.000 | 0.001 |

Appendix 1, Table 2. List of breeding bird species and their respective percent of the total captures (standardized by birds per year of effort) for each of the 25 stations in southern Oregon and northern California.

| Species | 7MIL | ANT1 | APRI | CABN | CAMP | CAPD | GBCR | GERB | GROV | HCME | HOME | JOHN | LADY | LELA | MARI | ODES | PARK | PCT1 | TOPS | WIIM | WILL | WIWI | WOOD | WREF | YACR |
| --- | --- | --- | --- | --- | --- | --- | --- | --- | --- | --- | --- | --- | --- | --- | --- | --- | --- | --- | --- | --- | --- | --- | --- | --- | --- |
| Acorn Woodpecker | 0.000 | 0.000 | 0.000 | 0.000 | 0.000 | 0.000 | 0.000 | 0.000 | 0.000 | 0.000 | 0.000 | 0.000 | 0.000 | 0.000 | 0.000 | 0.000 | 0.000 | 0.000 | 0.067 | 0.000 | 0.000 | 0.933 | 0.000 | 0.000 | 0.000 |
| Alder/Willow Flycatcher(Traill's) | 0.000 | 0.000 | 0.000 | 0.000 | 0.000 | 0.043 | 0.000 | 0.000 | 0.000 | 0.000 | 0.000 | 0.000 | 0.100 | 0.000 | 0.000 | 0.000 | 0.000 | 0.856 | 0.000 | 0.000 | 0.000 | 0.000 | 0.000 | 0.000 | 0.000 |
| Allen's Hummingbird | 0.010 | 0.009 | 0.000 | 0.000 | 0.085 | 0.093 | 0.000 | 0.000 | 0.039 | 0.000 | 0.452 | 0.000 | 0.048 | 0.078 | 0.012 | 0.000 | 0.058 | 0.019 | 0.011 | 0.000 | 0.022 | 0.000 | 0.000 | 0.000 | 0.062 |
| American Goldfinch | 0.006 | 0.000 | 0.008 | 0.001 | 0.000 | 0.000 | 0.000 | 0.000 | 0.000 | 0.000 | 0.454 | 0.000 | 0.001 | 0.102 | 0.008 | 0.000 | 0.097 | 0.006 | 0.006 | 0.003 | 0.000 | 0.145 | 0.157 | 0.003 | 0.003 |
| American Robin | 0.085 | 0.026 | 0.020 | 0.066 | 0.010 | 0.009 | 0.004 | 0.054 | 0.065 | 0.015 | 0.054 | 0.013 | 0.001 | 0.034 | 0.026 | 0.119 | 0.036 | 0.003 | 0.034 | 0.004 | 0.061 | 0.012 | 0.199 | 0.012 | 0.038 |
| Anna's Hummingbird | 0.000 | 0.000 | 0.000 | 0.000 | 0.000 | 0.000 | 0.000 | 0.000 | 0.000 | 0.000 | 0.129 | 0.000 | 0.000 | 0.000 | 0.000 | 0.000 | 0.000 | 0.000 | 0.000 | 0.158 | 0.000 | 0.712 | 0.000 | 0.000 | 0.000 |
| Ash-throated Flycatcher | 0.000 | 0.000 | 0.000 | 0.000 | 0.000 | 0.000 | 0.000 | 0.000 | 0.000 | 0.000 | 0.000 | 0.000 | 0.000 | 0.000 | 0.000 | 0.000 | 0.000 | 0.692 | 0.000 | 0.308 | 0.000 | 0.000 | 0.000 | 0.000 | 0.000 |
| Bewick's Wren | 0.000 | 0.000 | 0.018 | 0.000 | 0.000 | 0.000 | 0.000 | 0.000 | 0.000 | 0.011 | 0.036 | 0.000 | 0.000 | 0.079 | 0.000 | 0.000 | 0.000 | 0.221 | 0.014 | 0.011 | 0.000 | 0.394 | 0.000 | 0.000 | 0.217 |
| Black Phoebe | 0.000 | 0.000 | 0.000 | 0.000 | 0.000 | 0.000 | 0.000 | 0.000 | 0.000 | 0.000 | 0.479 | 0.000 | 0.000 | 0.000 | 0.000 | 0.000 | 0.000 | 0.101 | 0.000 | 0.045 | 0.000 | 0.000 | 0.000 | 0.051 | 0.324 |
| Black-capped Chickadee | 0.000 | 0.000 | 0.067 | 0.021 | 0.049 | 0.000 | 0.000 | 0.000 | 0.000 | 0.004 | 0.021 | 0.000 | 0.000 | 0.000 | 0.047 | 0.055 | 0.000 | 0.067 | 0.000 | 0.086 | 0.000 | 0.506 | 0.076 | 0.000 | 0.000 |
| Black-headed Grosbeak | 0.046 | 0.001 | 0.046 | 0.060 | 0.033 | 0.050 | 0.021 | 0.056 | 0.023 | 0.040 | 0.003 | 0.017 | 0.021 | 0.065 | 0.024 | 0.076 | 0.001 | 0.039 | 0.109 | 0.065 | 0.049 | 0.075 | 0.054 | 0.010 | 0.015 |
| Black-throated Gray Warbler | 0.000 | 0.000 | 0.000 | 0.000 | 0.000 | 0.000 | 0.461 | 0.000 | 0.000 | 0.088 | 0.000 | 0.000 | 0.000 | 0.190 | 0.049 | 0.000 | 0.000 | 0.099 | 0.000 | 0.018 | 0.000 | 0.000 | 0.000 | 0.000 | 0.095 |
| Brewer's Blackbird | 0.000 | 0.000 | 0.000 | 0.000 | 0.000 | 0.000 | 0.000 | 0.036 | 0.000 | 0.000 | 0.164 | 0.000 | 0.000 | 0.000 | 0.000 | 0.000 | 0.000 | 0.000 | 0.000 | 0.000 | 0.000 | 0.800 | 0.000 | 0.000 | 0.000 |
| Brewer's Sparrow | 0.247 | 0.109 | 0.000 | 0.000 | 0.000 | 0.000 | 0.000 | 0.169 | 0.000 | 0.103 | 0.000 | 0.124 | 0.000 | 0.000 | 0.000 | 0.116 | 0.000 | 0.000 | 0.000 | 0.000 | 0.132 | 0.000 | 0.000 | 0.000 | 0.000 |
| Brown Creeper | 0.084 | 0.011 | 0.106 | 0.024 | 0.000 | 0.000 | 0.000 | 0.132 | 0.006 | 0.000 | 0.000 | 0.071 | 0.000 | 0.000 | 0.000 | 0.170 | 0.006 | 0.000 | 0.035 | 0.022 | 0.076 | 0.000 | 0.000 | 0.091 | 0.165 |
| Brown-headed Cowbird | 0.000 | 0.000 | 0.000 | 0.073 | 0.000 | 0.000 | 0.000 | 0.000 | 0.000 | 0.000 | 0.107 | 0.000 | 0.000 | 0.000 | 0.000 | 0.000 | 0.000 | 0.000 | 0.000 | 0.000 | 0.000 | 0.586 | 0.234 | 0.000 | 0.000 |
| Bullock's Oriole | 0.004 | 0.000 | 0.045 | 0.013 | 0.000 | 0.002 | 0.000 | 0.000 | 0.000 | 0.000 | 0.106 | 0.000 | 0.002 | 0.035 | 0.000 | 0.020 | 0.013 | 0.011 | 0.144 | 0.010 | 0.000 | 0.562 | 0.035 | 0.000 | 0.000 |
| Bushtit | 0.000 | 0.000 | 0.016 | 0.000 | 0.000 | 0.000 | 0.023 | 0.047 | 0.005 | 0.067 | 0.020 | 0.000 | 0.000 | 0.060 | 0.000 | 0.032 | 0.005 | 0.075 | 0.086 | 0.010 | 0.018 | 0.474 | 0.000 | 0.000 | 0.060 |
| California Towhee | 0.000 | 0.000 | 0.000 | 0.000 | 0.000 | 0.000 | 0.000 | 0.000 | 0.000 | 0.000 | 0.000 | 0.000 | 0.000 | 0.000 | 0.000 | 0.000 | 0.000 | 0.000 | 0.000 | 0.000 | 0.000 | 0.909 | 0.000 | 0.000 | 0.091 |
| Calliope Hummingbird | 0.000 | 0.079 | 0.000 | 0.000 | 0.000 | 0.000 | 0.000 | 0.245 | 0.000 | 0.000 | 0.000 | 0.000 | 0.000 | 0.000 | 0.000 | 0.000 | 0.000 | 0.000 | 0.482 | 0.000 | 0.193 | 0.000 | 0.000 | 0.000 | 0.000 |
| Cassin's Finch | 0.056 | 0.122 | 0.000 | 0.052 | 0.000 | 0.000 | 0.000 | 0.038 | 0.000 | 0.000 | 0.000 | 0.000 | 0.000 | 0.000 | 0.000 | 0.078 | 0.000 | 0.000 | 0.624 | 0.000 | 0.030 | 0.000 | 0.000 | 0.000 | 0.000 |
| Cassin's Vireo | 0.022 | 0.043 | 0.007 | 0.061 | 0.037 | 0.011 | 0.007 | 0.037 | 0.051 | 0.091 | 0.000 | 0.011 | 0.006 | 0.025 | 0.000 | 0.041 | 0.000 | 0.005 | 0.385 | 0.014 | 0.099 | 0.000 | 0.005 | 0.000 | 0.041 |
| Cedar Waxwing | 0.000 | 0.000 | 0.000 | 0.014 | 0.000 | 0.000 | 0.000 | 0.000 | 0.000 | 0.000 | 0.281 | 0.000 | 0.000 | 0.046 | 0.203 | 0.050 | 0.072 | 0.036 | 0.000 | 0.032 | 0.008 | 0.000 | 0.038 | 0.014 | 0.206 |
| Chestnut-backed Chickadee | 0.035 | 0.000 | 0.000 | 0.027 | 0.000 | 0.000 | 0.016 | 0.000 | 0.082 | 0.029 | 0.150 | 0.023 | 0.000 | 0.165 | 0.127 | 0.000 | 0.098 | 0.000 | 0.000 | 0.000 | 0.000 | 0.000 | 0.000 | 0.022 | 0.226 |
| Chipping Sparrow | 0.145 | 0.026 | 0.000 | 0.068 | 0.000 | 0.015 | 0.000 | 0.178 | 0.014 | 0.000 | 0.000 | 0.015 | 0.000 | 0.000 | 0.000 | 0.000 | 0.000 | 0.014 | 0.249 | 0.012 | 0.265 | 0.000 | 0.000 | 0.000 | 0.000 |
| Common Yellowthroat | 0.014 | 0.000 | 0.000 | 0.146 | 0.000 | 0.000 | 0.000 | 0.000 | 0.000 | 0.000 | 0.019 | 0.000 | 0.000 | 0.000 | 0.065 | 0.266 | 0.000 | 0.000 | 0.015 | 0.000 | 0.000 | 0.106 | 0.354 | 0.013 | 0.000 |
| Dark-eyed Junco | 0.147 | 0.084 | 0.000 | 0.062 | 0.000 | 0.001 | 0.109 | 0.065 | 0.183 | 0.030 | 0.000 | 0.212 | 0.000 | 0.000 | 0.000 | 0.028 | 0.000 | 0.000 | 0.002 | 0.000 | 0.041 | 0.000 | 0.000 | 0.000 | 0.037 |
| Downy Woodpecker | 0.028 | 0.010 | 0.053 | 0.029 | 0.018 | 0.006 | 0.000 | 0.003 | 0.010 | 0.008 | 0.023 | 0.000 | 0.009 | 0.011 | 0.026 | 0.021 | 0.014 | 0.022 | 0.061 | 0.063 | 0.028 | 0.429 | 0.096 | 0.012 | 0.022 |
| Dusky Flycatcher | 0.007 | 0.000 | 0.000 | 0.277 | 0.000 | 0.007 | 0.000 | 0.029 | 0.482 | 0.000 | 0.000 | 0.056 | 0.016 | 0.000 | 0.000 | 0.092 | 0.000 | 0.000 | 0.008 | 0.000 | 0.015 | 0.000 | 0.000 | 0.000 | 0.011 |
| European Starling | 0.013 | 0.000 | 0.000 | 0.000 | 0.000 | 0.000 | 0.000 | 0.000 | 0.000 | 0.000 | 0.256 | 0.000 | 0.000 | 0.000 | 0.000 | 0.000 | 0.047 | 0.000 | 0.013 | 0.000 | 0.000 | 0.658 | 0.013 | 0.000 | 0.000 |
| Evening Grosbeak | 0.115 | 0.389 | 0.000 | 0.000 | 0.000 | 0.000 | 0.000 | 0.000 | 0.018 | 0.000 | 0.000 | 0.422 | 0.000 | 0.000 | 0.000 | 0.036 | 0.000 | 0.000 | 0.000 | 0.000 | 0.021 | 0.000 | 0.000 | 0.000 | 0.000 |
| Fox Sparrow | 0.000 | 0.025 | 0.000 | 0.026 | 0.000 | 0.000 | 0.000 | 0.000 | 0.132 | 0.000 | 0.019 | 0.000 | 0.000 | 0.000 | 0.000 | 0.026 | 0.000 | 0.000 | 0.030 | 0.047 | 0.694 | 0.000 | 0.000 | 0.000 | 0.000 |
| Golden-crowned Kinglet | 0.184 | 0.023 | 0.000 | 0.173 | 0.000 | 0.000 | 0.000 | 0.000 | 0.123 | 0.000 | 0.036 | 0.329 | 0.000 | 0.000 | 0.000 | 0.000 | 0.012 | 0.000 | 0.000 | 0.000 | 0.000 | 0.000 | 0.000 | 0.099 | 0.020 |
| Golden-crowned Sparrow | 0.000 | 0.000 | 0.000 | 0.000 | 0.000 | 0.000 | 0.000 | 0.000 | 0.000 | 0.000 | 0.421 | 0.000 | 0.000 | 0.000 | 0.000 | 0.000 | 0.000 | 0.579 | 0.000 | 0.000 | 0.000 | 0.000 | 0.000 | 0.000 | 0.000 |
| Gray Flycatcher | 0.000 | 0.087 | 0.000 | 0.093 | 0.000 | 0.000 | 0.000 | 0.541 | 0.279 | 0.000 | 0.000 | 0.000 | 0.000 | 0.000 | 0.000 | 0.000 | 0.000 | 0.000 | 0.000 | 0.000 | 0.000 | 0.000 | 0.000 | 0.000 | 0.000 |
| Gray Jay | 0.000 | 0.000 | 0.000 | 0.000 | 0.000 | 0.000 | 0.000 | 0.000 | 0.000 | 0.000 | 0.000 | 1.000 | 0.000 | 0.000 | 0.000 | 0.000 | 0.000 | 0.000 | 0.000 | 0.000 | 0.000 | 0.000 | 0.000 | 0.000 | 0.000 |
| Green-tailed Towhee | 0.000 | 0.000 | 0.000 | 0.000 | 0.000 | 0.000 | 0.000 | 0.066 | 0.000 | 0.000 | 0.000 | 0.049 | 0.000 | 0.000 | 0.000 | 0.000 | 0.000 | 0.000 | 0.052 | 0.000 | 0.833 | 0.000 | 0.000 | 0.000 | 0.000 |
| Hairy Woodpecker | 0.114 | 0.022 | 0.104 | 0.000 | 0.000 | 0.013 | 0.069 | 0.069 | 0.012 | 0.032 | 0.026 | 0.000 | 0.000 | 0.076 | 0.161 | 0.060 | 0.060 | 0.024 | 0.000 | 0.011 | 0.054 | 0.000 | 0.000 | 0.036 | 0.057 |
| Hammond's Flycatcher | 0.085 | 0.038 | 0.000 | 0.080 | 0.000 | 0.014 | 0.000 | 0.019 | 0.239 | 0.000 | 0.000 | 0.213 | 0.000 | 0.000 | 0.000 | 0.053 | 0.000 | 0.000 | 0.015 | 0.000 | 0.030 | 0.213 | 0.000 | 0.000 | 0.000 |
| Hermit Thrush | 0.028 | 0.005 | 0.000 | 0.144 | 0.023 | 0.023 | 0.248 | 0.000 | 0.096 | 0.057 | 0.000 | 0.159 | 0.013 | 0.000 | 0.000 | 0.043 | 0.000 | 0.032 | 0.006 | 0.000 | 0.030 | 0.043 | 0.006 | 0.000 | 0.043 |
| Hermit Warbler | 0.050 | 0.079 | 0.000 | 0.236 | 0.000 | 0.000 | 0.064 | 0.000 | 0.192 | 0.072 | 0.000 | 0.223 | 0.000 | 0.000 | 0.000 | 0.041 | 0.000 | 0.000 | 0.000 | 0.000 | 0.004 | 0.000 | 0.000 | 0.000 | 0.038 |
| House Finch | 0.000 | 0.000 | 0.009 | 0.000 | 0.000 | 0.000 | 0.000 | 0.000 | 0.000 | 0.000 | 0.541 | 0.000 | 0.000 | 0.010 | 0.000 | 0.000 | 0.000 | 0.000 | 0.000 | 0.000 | 0.000 | 0.432 | 0.000 | 0.000 | 0.010 |
| House Sparrow | 0.000 | 0.000 | 0.000 | 0.000 | 0.000 | 0.000 | 0.000 | 0.000 | 0.000 | 0.000 | 0.015 | 0.000 | 0.000 | 0.000 | 0.000 | 0.000 | 0.000 | 0.000 | 0.000 | 0.009 | 0.000 | 0.976 | 0.000 | 0.000 | 0.000 |
| House Wren | 0.418 | 0.000 | 0.216 | 0.000 | 0.000 | 0.000 | 0.000 | 0.015 | 0.000 | 0.066 | 0.000 | 0.000 | 0.000 | 0.000 | 0.000 | 0.159 | 0.000 | 0.021 | 0.048 | 0.000 | 0.012 | 0.000 | 0.045 | 0.000 | 0.000 |
| Hutton's Vireo | 0.000 | 0.000 | 0.000 | 0.000 | 0.029 | 0.021 | 0.088 | 0.000 | 0.020 | 0.072 | 0.146 | 0.000 | 0.000 | 0.000 | 0.000 | 0.000 | 0.040 | 0.000 | 0.000 | 0.000 | 0.000 | 0.000 | 0.000 | 0.101 | 0.483 |
| Lazuli Bunting | 0.200 | 0.097 | 0.022 | 0.008 | 0.002 | 0.010 | 0.000 | 0.009 | 0.087 | 0.070 | 0.003 | 0.136 | 0.051 | 0.002 | 0.000 | 0.009 | 0.000 | 0.014 | 0.191 | 0.015 | 0.005 | 0.049 | 0.002 | 0.009 | 0.010 |
| Lesser Goldfinch | 0.002 | 0.000 | 0.080 | 0.000 | 0.002 | 0.004 | 0.000 | 0.000 | 0.000 | 0.017 | 0.001 | 0.000 | 0.004 | 0.025 | 0.000 | 0.002 | 0.000 | 0.082 | 0.286 | 0.002 | 0.000 | 0.479 | 0.004 | 0.009 | 0.003 |
| Lincoln's Sparrow | 0.122 | 0.463 | 0.000 | 0.000 | 0.006 | 0.000 | 0.000 | 0.000 | 0.348 | 0.000 | 0.000 | 0.045 | 0.005 | 0.000 | 0.010 | 0.000 | 0.000 | 0.000 | 0.000 | 0.000 | 0.000 | 0.000 | 0.000 | 0.000 | 0.000 |
| MacGillivray's Warbler | 0.166 | 0.042 | 0.012 | 0.006 | 0.009 | 0.045 | 0.084 | 0.001 | 0.060 | 0.114 | 0.000 | 0.099 | 0.031 | 0.000 | 0.103 | 0.025 | 0.000 | 0.107 | 0.006 | 0.033 | 0.046 | 0.010 | 0.001 | 0.000 | 0.000 |
| Marsh Wren | 0.000 | 0.000 | 0.000 | 0.246 | 0.000 | 0.000 | 0.000 | 0.000 | 0.000 | 0.000 | 0.000 | 0.000 | 0.000 | 0.000 | 0.000 | 0.246 | 0.000 | 0.000 | 0.000 | 0.000 | 0.000 | 0.000 | 0.262 | 0.246 | 0.000 |
| Mountain Chickadee | 0.107 | 0.084 | 0.000 | 0.061 | 0.000 | 0.000 | 0.000 | 0.382 | 0.000 | 0.000 | 0.000 | 0.042 | 0.000 | 0.000 | 0.000 | 0.101 | 0.000 | 0.000 | 0.000 | 0.000 | 0.211 | 0.000 | 0.012 | 0.000 | 0.000 |
| Mourning Dove | 0.000 | 0.000 | 0.000 | 0.000 | 0.000 | 0.000 | 0.000 | 0.000 | 0.000 | 0.000 | 0.577 | 0.000 | 0.000 | 0.000 | 0.000 | 0.000 | 0.000 | 0.000 | 0.000 | 0.000 | 0.000 | 0.000 | 0.423 | 0.000 | 0.000 |
| Nashville Warbler | 0.234 | 0.095 | 0.003 | 0.034 | 0.003 | 0.012 | 0.184 | 0.008 | 0.015 | 0.134 | 0.001 | 0.135 | 0.002 | 0.000 | 0.000 | 0.034 | 0.000 | 0.032 | 0.040 | 0.003 | 0.023 | 0.000 | 0.008 | 0.000 | 0.000 |
| Northern Flicker | 0.038 | 0.007 | 0.010 | 0.106 | 0.061 | 0.000 | 0.000 | 0.072 | 0.042 | 0.000 | 0.102 | 0.000 | 0.000 | 0.000 | 0.000 | 0.056 | 0.007 | 0.028 | 0.016 | 0.025 | 0.016 | 0.394 | 0.008 | 0.000 | 0.011 |
| Oak Titmouse | 0.000 | 0.000 | 0.000 | 0.000 | 0.000 | 0.000 | 0.000 | 0.000 | 0.000 | 0.000 | 0.000 | 0.000 | 0.000 | 0.000 | 0.000 | 0.000 | 0.000 | 0.000 | 1.000 | 0.000 | 0.000 | 0.000 | 0.000 | 0.000 | 0.000 |
| Olive-sided Flycatcher | 0.000 | 0.072 | 0.000 | 0.077 | 0.000 | 0.000 | 0.000 | 0.000 | 0.000 | 0.000 | 0.446 | 0.000 | 0.000 | 0.000 | 0.000 | 0.153 | 0.000 | 0.077 | 0.000 | 0.000 | 0.175 | 0.000 | 0.000 | 0.000 | 0.000 |
| Orange-crowned Warbler | 0.026 | 0.032 | 0.018 | 0.012 | 0.046 | 0.040 | 0.000 | 0.021 | 0.018 | 0.086 | 0.105 | 0.008 | 0.050 | 0.025 | 0.039 | 0.011 | 0.048 | 0.004 | 0.008 | 0.116 | 0.036 | 0.014 | 0.006 | 0.106 | 0.125 |
| Pacific-slope/Cordilleran Flycatcher | 0.013 | 0.007 | 0.036 | 0.007 | 0.025 | 0.005 | 0.029 | 0.021 | 0.020 | 0.026 | 0.125 | 0.013 | 0.060 | 0.047 | 0.082 | 0.007 | 0.093 | 0.007 | 0.003 | 0.031 | 0.000 | 0.039 | 0.000 | 0.194 | 0.110 |
| Pacific Wren | 0.000 | 0.000 | 0.000 | 0.000 | 0.000 | 0.000 | 0.000 | 0.000 | 0.015 | 0.000 | 0.128 | 0.016 | 0.036 | 0.070 | 0.018 | 0.000 | 0.044 | 0.000 | 0.000 | 0.000 | 0.000 | 0.000 | 0.000 | 0.674 | 0.000 |
| Pileated Woodpecker | 0.000 | 0.000 | 0.000 | 0.000 | 0.000 | 0.000 | 1.000 | 0.000 | 0.000 | 0.000 | 0.000 | 0.000 | 0.000 | 0.000 | 0.000 | 0.000 | 0.000 | 0.000 | 0.000 | 0.000 | 0.000 | 0.000 | 0.000 | 0.000 | 0.000 |
| Pine Siskin | 0.026 | 0.048 | 0.000 | 0.085 | 0.000 | 0.000 | 0.000 | 0.000 | 0.079 | 0.030 | 0.493 | 0.036 | 0.000 | 0.000 | 0.000 | 0.009 | 0.033 | 0.000 | 0.007 | 0.000 | 0.003 | 0.145 | 0.006 | 0.000 | 0.000 |
| Purple Finch | 0.002 | 0.003 | 0.003 | 0.017 | 0.004 | 0.007 | 0.002 | 0.000 | 0.034 | 0.068 | 0.243 | 0.002 | 0.003 | 0.005 | 0.015 | 0.030 | 0.034 | 0.038 | 0.398 | 0.009 | 0.034 | 0.031 | 0.010 | 0.005 | 0.004 |
| Pygmy Nuthatch | 0.000 | 0.000 | 0.000 | 0.000 | 0.000 | 0.000 | 0.000 | 0.322 | 0.000 | 0.000 | 0.000 | 0.000 | 0.000 | 0.000 | 0.000 | 0.442 | 0.000 | 0.000 | 0.000 | 0.000 | 0.000 | 0.000 | 0.236 | 0.000 | 0.000 |
| Red Crossbill | 0.000 | 0.000 | 0.000 | 0.000 | 0.000 | 0.000 | 0.000 | 0.000 | 0.097 | 0.000 | 0.708 | 0.000 | 0.000 | 0.000 | 0.000 | 0.000 | 0.195 | 0.000 | 0.000 | 0.000 | 0.000 | 0.000 | 0.000 | 0.000 | 0.000 |
| Red-breasted Nuthatch | 0.063 | 0.037 | 0.000 | 0.217 | 0.000 | 0.000 | 0.000 | 0.086 | 0.079 | 0.000 | 0.014 | 0.253 | 0.000 | 0.000 | 0.000 | 0.138 | 0.000 | 0.000 | 0.023 | 0.000 | 0.090 | 0.000 | 0.000 | 0.000 | 0.000 |
| Red-breasted Sapsucker | 0.173 | 0.092 | 0.024 | 0.067 | 0.015 | 0.002 | 0.000 | 0.180 | 0.053 | 0.016 | 0.000 | 0.011 | 0.000 | 0.000 | 0.007 | 0.108 | 0.000 | 0.026 | 0.016 | 0.087 | 0.100 | 0.000 | 0.017 | 0.000 | 0.006 |
| Red-naped Sapsucker | 0.592 | 0.000 | 0.000 | 0.000 | 0.000 | 0.000 | 0.000 | 0.000 | 0.000 | 0.000 | 0.000 | 0.000 | 0.000 | 0.000 | 0.000 | 0.000 | 0.000 | 0.000 | 0.211 | 0.000 | 0.000 | 0.000 | 0.197 | 0.000 | 0.000 |
| Red-winged Blackbird | 0.000 | 0.000 | 0.000 | 0.113 | 0.000 | 0.000 | 0.000 | 0.000 | 0.000 | 0.000 | 0.000 | 0.000 | 0.000 | 0.000 | 0.000 | 0.051 | 0.000 | 0.001 | 0.002 | 0.000 | 0.000 | 0.676 | 0.156 | 0.000 | 0.000 |
| Ruffed Grouse | 0.000 | 0.000 | 0.000 | 1.000 | 0.000 | 0.000 | 0.000 | 0.000 | 0.000 | 0.000 | 0.000 | 0.000 | 0.000 | 0.000 | 0.000 | 0.000 | 0.000 | 0.000 | 0.000 | 0.000 | 0.000 | 0.000 | 0.000 | 0.000 | 0.000 |
| Rufous Hummingbird | 0.065 | 0.019 | 0.015 | 0.071 | 0.015 | 0.011 | 0.103 | 0.030 | 0.020 | 0.072 | 0.015 | 0.032 | 0.012 | 0.000 | 0.000 | 0.000 | 0.000 | 0.304 | 0.035 | 0.099 | 0.000 | 0.081 | 0.000 | 0.000 | 0.000 |
| Savannah Sparrow | 0.000 | 0.000 | 0.000 | 0.000 | 0.000 | 0.000 | 0.000 | 0.000 | 0.000 | 0.000 | 1.000 | 0.000 | 0.000 | 0.000 | 0.000 | 0.000 | 0.000 | 0.000 | 0.000 | 0.000 | 0.000 | 0.000 | 0.000 | 0.000 | 0.000 |
| Song Sparrow | 0.020 | 0.010 | 0.050 | 0.054 | 0.008 | 0.008 | 0.003 | 0.006 | 0.041 | 0.016 | 0.041 | 0.008 | 0.018 | 0.046 | 0.044 | 0.048 | 0.044 | 0.034 | 0.032 | 0.045 | 0.045 | 0.188 | 0.123 | 0.020 | 0.049 |
| Spotted Towhee | 0.005 | 0.000 | 0.120 | 0.001 | 0.076 | 0.102 | 0.051 | 0.053 | 0.003 | 0.069 | 0.000 | 0.000 | 0.060 | 0.000 | 0.000 | 0.035 | 0.000 | 0.118 | 0.043 | 0.067 | 0.034 | 0.160 | 0.002 | 0.000 | 0.003 |
| Steller's Jay | 0.032 | 0.056 | 0.000 | 0.030 | 0.000 | 0.127 | 0.043 | 0.000 | 0.089 | 0.106 | 0.000 | 0.032 | 0.000 | 0.000 | 0.073 | 0.030 | 0.000 | 0.000 | 0.034 | 0.000 | 0.034 | 0.000 | 0.000 | 0.030 | 0.285 |
| Swainson's Thrush | 0.002 | 0.000 | 0.002 | 0.001 | 0.033 | 0.011 | 0.035 | 0.000 | 0.006 | 0.006 | 0.110 | 0.005 | 0.033 | 0.201 | 0.175 | 0.013 | 0.091 | 0.007 | 0.001 | 0.008 | 0.001 | 0.011 | 0.001 | 0.126 | 0.121 |
| Townsend's Solitaire | 0.000 | 0.000 | 0.000 | 0.000 | 0.000 | 0.000 | 0.000 | 0.495 | 0.068 | 0.000 | 0.000 | 0.000 | 0.000 | 0.000 | 0.000 | 0.204 | 0.000 | 0.000 | 0.000 | 0.000 | 0.233 | 0.000 | 0.000 | 0.000 | 0.000 |
| Townsend's Warbler | 0.244 | 0.000 | 0.000 | 0.000 | 0.000 | 0.000 | 0.000 | 0.111 | 0.076 | 0.000 | 0.000 | 0.244 | 0.000 | 0.000 | 0.000 | 0.076 | 0.000 | 0.000 | 0.000 | 0.000 | 0.087 | 0.000 | 0.162 | 0.000 | 0.000 |
| Varied Thrush | 0.000 | 0.000 | 0.000 | 0.000 | 0.000 | 0.000 | 0.000 | 0.000 | 0.000 | 0.000 | 1.000 | 0.000 | 0.000 | 0.000 | 0.000 | 0.000 | 0.000 | 0.000 | 0.000 | 0.000 | 0.000 | 0.000 | 0.000 | 0.000 | 0.000 |
| Vesper Sparrow | 0.000 | 0.000 | 0.000 | 0.000 | 0.000 | 0.000 | 0.000 | 1.000 | 0.000 | 0.000 | 0.000 | 0.000 | 0.000 | 0.000 | 0.000 | 0.000 | 0.000 | 0.000 | 0.000 | 0.000 | 0.000 | 0.000 | 0.000 | 0.000 | 0.000 |
| Warbling Vireo | 0.082 | 0.062 | 0.017 | 0.073 | 0.000 | 0.000 | 0.055 | 0.017 | 0.082 | 0.158 | 0.002 | 0.105 | 0.000 | 0.045 | 0.055 | 0.030 | 0.002 | 0.005 | 0.166 | 0.010 | 0.024 | 0.000 | 0.010 | 0.000 | 0.000 |
| Western Bluebird | 0.000 | 0.000 | 0.593 | 0.000 | 0.000 | 0.000 | 0.000 | 0.000 | 0.407 | 0.000 | 0.000 | 0.000 | 0.000 | 0.000 | 0.000 | 0.000 | 0.000 | 0.000 | 0.000 | 0.000 | 0.000 | 0.000 | 0.000 | 0.000 | 0.000 |
| Western Scrub-Jay | 0.000 | 0.000 | 0.040 | 0.000 | 0.061 | 0.148 | 0.000 | 0.000 | 0.000 | 0.000 | 0.000 | 0.000 | 0.017 | 0.000 | 0.000 | 0.000 | 0.000 | 0.042 | 0.000 | 0.025 | 0.000 | 0.667 | 0.000 | 0.000 | 0.000 |
| Western Tanager | 0.037 | 0.013 | 0.000 | 0.349 | 0.018 | 0.064 | 0.011 | 0.022 | 0.014 | 0.024 | 0.000 | 0.097 | 0.006 | 0.000 | 0.002 | 0.043 | 0.000 | 0.036 | 0.173 | 0.009 | 0.033 | 0.030 | 0.012 | 0.000 | 0.010 |
| Western Wood-Pewee | 0.077 | 0.020 | 0.096 | 0.159 | 0.000 | 0.000 | 0.000 | 0.013 | 0.006 | 0.003 | 0.009 | 0.000 | 0.004 | 0.058 | 0.000 | 0.135 | 0.018 | 0.009 | 0.103 | 0.003 | 0.055 | 0.072 | 0.128 | 0.000 | 0.034 |
| White-breasted Nuthatch | 0.000 | 0.000 | 0.000 | 0.000 | 0.000 | 0.000 | 0.000 | 0.241 | 0.000 | 0.000 | 0.000 | 0.000 | 0.000 | 0.000 | 0.000 | 0.000 | 0.000 | 0.000 | 0.284 | 0.000 | 0.474 | 0.000 | 0.000 | 0.000 | 0.000 |
| White-crowned Sparrow | 0.000 | 0.000 | 0.000 | 0.000 | 0.000 | 0.000 | 0.000 | 0.000 | 0.000 | 0.203 | 0.518 | 0.000 | 0.000 | 0.025 | 0.000 | 0.000 | 0.172 | 0.000 | 0.000 | 0.000 | 0.000 | 0.000 | 0.000 | 0.016 | 0.067 |
| White-headed Woodpecker | 0.000 | 0.239 | 0.000 | 0.761 | 0.000 | 0.000 | 0.000 | 0.000 | 0.000 | 0.000 | 0.000 | 0.000 | 0.000 | 0.000 | 0.000 | 0.000 | 0.000 | 0.000 | 0.000 | 0.000 | 0.000 | 0.000 | 0.000 | 0.000 | 0.000 |
| Williamson's Sapsucker | 0.000 | 0.000 | 0.000 | 0.429 | 0.000 | 0.000 | 0.000 | 0.000 | 0.000 | 0.000 | 0.000 | 0.000 | 0.000 | 0.000 | 0.000 | 0.571 | 0.000 | 0.000 | 0.000 | 0.000 | 0.000 | 0.000 | 0.000 | 0.000 | 0.000 |
| Willow Flycatcher | 0.078 | 0.000 | 0.030 | 0.219 | 0.000 | 0.011 | 0.000 | 0.030 | 0.021 | 0.019 | 0.008 | 0.022 | 0.000 | 0.050 | 0.000 | 0.042 | 0.000 | 0.063 | 0.024 | 0.083 | 0.000 | 0.167 | 0.134 | 0.000 | 0.000 |
| Wilson's Warbler | 0.004 | 0.204 | 0.001 | 0.003 | 0.003 | 0.013 | 0.003 | 0.000 | 0.026 | 0.012 | 0.038 | 0.066 | 0.014 | 0.071 | 0.138 | 0.008 | 0.033 | 0.012 | 0.007 | 0.003 | 0.003 | 0.008 | 0.002 | 0.224 | 0.102 |
| Wrentit | 0.000 | 0.000 | 0.078 | 0.000 | 0.133 | 0.053 | 0.000 | 0.000 | 0.001 | 0.012 | 0.127 | 0.000 | 0.090 | 0.049 | 0.063 | 0.000 | 0.072 | 0.008 | 0.000 | 0.115 | 0.000 | 0.085 | 0.000 | 0.049 | 0.064 |
| Yellow Warbler | 0.011 | 0.001 | 0.118 | 0.052 | 0.009 | 0.004 | 0.000 | 0.001 | 0.000 | 0.001 | 0.002 | 0.001 | 0.066 | 0.007 | 0.002 | 0.161 | 0.000 | 0.107 | 0.153 | 0.018 | 0.031 | 0.028 | 0.228 | 0.000 | 0.000 |
| Yellow-breasted Chat | 0.000 | 0.000 | 0.098 | 0.000 | 0.101 | 0.059 | 0.000 | 0.000 | 0.000 | 0.001 | 0.000 | 0.000 | 0.154 | 0.032 | 0.026 | 0.000 | 0.003 | 0.229 | 0.022 | 0.102 | 0.000 | 0.171 | 0.000 | 0.000 | 0.004 |
| Yellow-headed Blackbird | 0.000 | 0.000 | 0.000 | 0.000 | 0.000 | 0.000 | 0.000 | 0.000 | 0.000 | 0.000 | 0.000 | 0.000 | 0.000 | 0.000 | 0.000 | 0.000 | 0.000 | 0.000 | 0.000 | 0.000 | 0.000 | 0.000 | 1.000 | 0.000 | 0.000 |
| Yellow-rumped Warbler | 0.068 | 0.094 | 0.000 | 0.199 | 0.000 | 0.000 | 0.000 | 0.013 | 0.093 | 0.004 | 0.032 | 0.136 | 0.000 | 0.000 | 0.000 | 0.081 | 0.004 | 0.001 | 0.203 | 0.000 | 0.064 | 0.000 | 0.008 | 0.000 | 0.000 |

Appendix 1, Table 3. List of molting bird species and their respective percent of the total captures (standardized by birds per year of effort) for each of the 25 stations southern Oregon and northern California.

| Species | 7MIL | ANT1 | APRI | CABN | CAMP | CAPD | GBCR | GERB | GROV | HCME | HOME | JOHN | LADY | LELA | MARI | ODES | PARK | PCT1 | TOPS | WIIM | WILL | WIWI | WOOD | WREF | YACR |
| --- | --- | --- | --- | --- | --- | --- | --- | --- | --- | --- | --- | --- | --- | --- | --- | --- | --- | --- | --- | --- | --- | --- | --- | --- | --- |
| Acorn Woodpecker | 0.000 | 0.000 | 0.000 | 0.000 | 0.000 | 0.000 | 0.000 | 0.000 | 0.000 | 0.000 | 0.000 | 0.000 | 0.000 | 0.000 | 0.000 | 0.000 | 0.000 | 0.000 | 1.000 | 0.000 | 0.000 | 0.000 | 0.000 | 0.000 | 0.000 |
| Alder/Willow Flycatcher(Traill's) | 0.000 | 0.000 | 0.000 | 0.000 | 0.000 | 0.000 | 0.000 | 0.000 | 0.000 | 0.000 | 0.000 | 0.000 | 0.000 | 0.000 | 0.000 | 0.000 | 0.000 | 1.000 | 0.000 | 0.000 | 0.000 | 0.000 | 0.000 | 0.000 | 0.000 |
| Allen's Hummingbird | 0.000 | 0.000 | 0.000 | 0.000 | 0.593 | 0.000 | 0.000 | 0.000 | 0.407 | 0.000 | 0.000 | 0.000 | 0.000 | 0.000 | 0.000 | 0.000 | 0.000 | 0.000 | 0.000 | 0.000 | 0.000 | 0.000 | 0.000 | 0.000 | 0.000 |
| American Goldfinch | 0.001 | 0.000 | 0.000 | 0.000 | 0.000 | 0.000 | 0.000 | 0.000 | 0.000 | 0.001 | 0.041 | 0.000 | 0.000 | 0.003 | 0.000 | 0.000 | 0.006 | 0.001 | 0.001 | 0.000 | 0.000 | 0.940 | 0.007 | 0.000 | 0.000 |
| American Robin | 0.008 | 0.036 | 0.082 | 0.033 | 0.034 | 0.014 | 0.007 | 0.048 | 0.018 | 0.009 | 0.061 | 0.027 | 0.000 | 0.012 | 0.006 | 0.154 | 0.038 | 0.008 | 0.018 | 0.036 | 0.173 | 0.000 | 0.156 | 0.005 | 0.016 |
| Anna's Hummingbird | 0.000 | 0.000 | 0.000 | 0.000 | 0.000 | 0.000 | 0.000 | 0.000 | 0.000 | 0.000 | 0.047 | 0.000 | 0.027 | 0.000 | 0.000 | 0.000 | 0.022 | 0.000 | 0.000 | 0.000 | 0.000 | 0.869 | 0.000 | 0.000 | 0.035 |
| Bewick's Wren | 0.000 | 0.015 | 0.000 | 0.000 | 0.000 | 0.000 | 0.000 | 0.000 | 0.000 | 0.028 | 0.000 | 0.000 | 0.000 | 0.050 | 0.000 | 0.000 | 0.000 | 0.125 | 0.018 | 0.000 | 0.000 | 0.748 | 0.017 | 0.000 | 0.000 |
| Black Phoebe | 0.000 | 0.000 | 0.000 | 0.000 | 0.099 | 0.072 | 0.000 | 0.000 | 0.000 | 0.000 | 0.542 | 0.000 | 0.083 | 0.000 | 0.000 | 0.000 | 0.136 | 0.068 | 0.000 | 0.000 | 0.000 | 0.000 | 0.000 | 0.000 | 0.000 |
| Black-capped Chickadee | 0.005 | 0.000 | 0.100 | 0.069 | 0.069 | 0.000 | 0.031 | 0.000 | 0.000 | 0.004 | 0.009 | 0.000 | 0.016 | 0.000 | 0.000 | 0.060 | 0.009 | 0.039 | 0.000 | 0.134 | 0.000 | 0.345 | 0.110 | 0.000 | 0.000 |
| Black-headed Grosbeak | 0.090 | 0.000 | 0.000 | 0.000 | 0.246 | 0.090 | 0.123 | 0.000 | 0.000 | 0.000 | 0.000 | 0.000 | 0.000 | 0.000 | 0.000 | 0.000 | 0.000 | 0.169 | 0.193 | 0.000 | 0.000 | 0.000 | 0.090 | 0.000 | 0.000 |
| Black-throated Gray Warbler | 0.000 | 0.000 | 0.000 | 0.000 | 0.000 | 0.000 | 0.142 | 0.000 | 0.000 | 0.348 | 0.000 | 0.000 | 0.000 | 0.078 | 0.000 | 0.000 | 0.000 | 0.245 | 0.056 | 0.131 | 0.000 | 0.000 | 0.000 | 0.000 | 0.000 |
| Brewer's Blackbird | 0.000 | 0.000 | 0.000 | 0.000 | 0.000 | 0.000 | 0.000 | 0.000 | 0.000 | 0.000 | 0.000 | 0.000 | 0.000 | 0.000 | 0.000 | 0.000 | 0.000 | 0.000 | 0.000 | 0.000 | 0.000 | 1.000 | 0.000 | 0.000 | 0.000 |
| Brewer's Sparrow | 0.083 | 0.018 | 0.000 | 0.469 | 0.000 | 0.000 | 0.000 | 0.028 | 0.000 | 0.000 | 0.000 | 0.021 | 0.000 | 0.000 | 0.000 | 0.117 | 0.000 | 0.039 | 0.000 | 0.000 | 0.067 | 0.156 | 0.000 | 0.000 | 0.000 |
| Brown Creeper | 0.087 | 0.038 | 0.148 | 0.142 | 0.000 | 0.000 | 0.000 | 0.148 | 0.000 | 0.000 | 0.000 | 0.000 | 0.000 | 0.000 | 0.000 | 0.264 | 0.000 | 0.000 | 0.070 | 0.000 | 0.070 | 0.000 | 0.000 | 0.000 | 0.033 |
| Brown-headed Cowbird | 0.000 | 0.000 | 0.000 | 0.000 | 0.000 | 0.000 | 0.000 | 0.000 | 0.000 | 0.000 | 0.000 | 0.000 | 0.000 | 0.000 | 0.000 | 0.000 | 0.000 | 0.000 | 0.000 | 0.000 | 0.000 | 0.000 | 1.000 | 0.000 | 0.000 |
| Bullock's Oriole | 0.000 | 0.000 | 0.000 | 0.000 | 0.000 | 0.000 | 0.000 | 0.000 | 0.000 | 0.000 | 0.000 | 0.000 | 0.000 | 0.000 | 0.000 | 0.467 | 0.000 | 0.000 | 0.533 | 0.000 | 0.000 | 0.000 | 0.000 | 0.000 | 0.000 |
| Bushtit | 0.000 | 0.000 | 0.013 | 0.018 | 0.007 | 0.019 | 0.020 | 0.040 | 0.000 | 0.040 | 0.013 | 0.000 | 0.000 | 0.007 | 0.006 | 0.014 | 0.000 | 0.086 | 0.098 | 0.028 | 0.026 | 0.435 | 0.000 | 0.000 | 0.130 |
| California Quail | 0.000 | 0.000 | 0.000 | 0.000 | 0.054 | 0.000 | 0.000 | 0.000 | 0.000 | 0.000 | 0.482 | 0.000 | 0.045 | 0.000 | 0.000 | 0.000 | 0.000 | 0.000 | 0.000 | 0.065 | 0.000 | 0.295 | 0.000 | 0.000 | 0.059 |
| California Towhee | 0.000 | 0.000 | 0.000 | 0.000 | 0.000 | 0.000 | 0.000 | 0.000 | 0.000 | 0.000 | 0.000 | 0.000 | 0.000 | 0.000 | 0.000 | 0.000 | 0.000 | 0.000 | 0.000 | 0.000 | 0.000 | 1.000 | 0.000 | 0.000 | 0.000 |
| Cassin's Finch | 0.038 | 0.134 | 0.000 | 0.000 | 0.000 | 0.000 | 0.000 | 0.155 | 0.000 | 0.032 | 0.000 | 0.000 | 0.000 | 0.000 | 0.000 | 0.071 | 0.000 | 0.000 | 0.285 | 0.000 | 0.285 | 0.000 | 0.000 | 0.000 | 0.000 |
| Cassin's Vireo | 0.018 | 0.081 | 0.004 | 0.096 | 0.057 | 0.008 | 0.007 | 0.039 | 0.020 | 0.046 | 0.002 | 0.024 | 0.006 | 0.000 | 0.003 | 0.039 | 0.000 | 0.022 | 0.361 | 0.039 | 0.107 | 0.020 | 0.000 | 0.000 | 0.000 |
| Cedar Waxwing | 0.000 | 0.000 | 0.066 | 0.000 | 0.000 | 0.000 | 0.000 | 0.000 | 0.000 | 0.020 | 0.099 | 0.000 | 0.083 | 0.000 | 0.000 | 0.000 | 0.023 | 0.023 | 0.000 | 0.663 | 0.000 | 0.000 | 0.024 | 0.000 | 0.000 |
| Chestnut-backed Chickadee | 0.029 | 0.000 | 0.000 | 0.171 | 0.013 | 0.000 | 0.013 | 0.000 | 0.036 | 0.032 | 0.216 | 0.058 | 0.000 | 0.115 | 0.022 | 0.000 | 0.036 | 0.000 | 0.000 | 0.000 | 0.000 | 0.000 | 0.000 | 0.027 | 0.231 |
| Chipping Sparrow | 0.027 | 0.000 | 0.000 | 0.126 | 0.000 | 0.013 | 0.000 | 0.532 | 0.000 | 0.000 | 0.000 | 0.013 | 0.000 | 0.000 | 0.000 | 0.000 | 0.000 | 0.000 | 0.029 | 0.000 | 0.259 | 0.000 | 0.000 | 0.000 | 0.000 |
| Common Yellowthroat | 0.000 | 0.000 | 0.000 | 0.000 | 0.000 | 0.000 | 0.000 | 0.000 | 0.000 | 0.000 | 0.000 | 0.000 | 0.000 | 0.000 | 0.000 | 0.142 | 0.000 | 0.000 | 0.023 | 0.000 | 0.000 | 0.488 | 0.347 | 0.000 | 0.000 |
| Dark-eyed Junco | 0.136 | 0.099 | 0.000 | 0.167 | 0.000 | 0.003 | 0.023 | 0.112 | 0.168 | 0.028 | 0.001 | 0.155 | 0.000 | 0.000 | 0.000 | 0.047 | 0.000 | 0.000 | 0.000 | 0.000 | 0.046 | 0.000 | 0.000 | 0.000 | 0.014 |
| Downy Woodpecker | 0.005 | 0.009 | 0.034 | 0.037 | 0.014 | 0.035 | 0.007 | 0.034 | 0.009 | 0.004 | 0.034 | 0.000 | 0.011 | 0.045 | 0.023 | 0.009 | 0.061 | 0.033 | 0.027 | 0.075 | 0.021 | 0.374 | 0.040 | 0.000 | 0.060 |
| Dusky Flycatcher | 0.000 | 0.188 | 0.000 | 0.499 | 0.000 | 0.000 | 0.000 | 0.000 | 0.100 | 0.000 | 0.000 | 0.213 | 0.000 | 0.000 | 0.000 | 0.000 | 0.000 | 0.000 | 0.000 | 0.000 | 0.000 | 0.000 | 0.000 | 0.000 | 0.000 |
| European Starling | 0.000 | 0.000 | 0.000 | 0.000 | 0.000 | 0.000 | 0.000 | 0.000 | 0.000 | 0.000 | 0.304 | 0.000 | 0.000 | 0.000 | 0.000 | 0.000 | 0.139 | 0.000 | 0.000 | 0.000 | 0.000 | 0.557 | 0.000 | 0.000 | 0.000 |
| Evening Grosbeak | 0.063 | 0.361 | 0.000 | 0.000 | 0.000 | 0.000 | 0.000 | 0.000 | 0.000 | 0.000 | 0.000 | 0.283 | 0.000 | 0.000 | 0.000 | 0.059 | 0.000 | 0.000 | 0.000 | 0.000 | 0.202 | 0.000 | 0.031 | 0.000 | 0.000 |
| Fox Sparrow | 0.000 | 0.173 | 0.000 | 0.183 | 0.000 | 0.000 | 0.000 | 0.000 | 0.000 | 0.000 | 0.133 | 0.000 | 0.000 | 0.000 | 0.000 | 0.000 | 0.061 | 0.000 | 0.000 | 0.109 | 0.280 | 0.000 | 0.000 | 0.061 | 0.000 |
| Golden-crowned Kinglet | 0.065 | 0.000 | 0.000 | 0.429 | 0.000 | 0.000 | 0.000 | 0.000 | 0.061 | 0.000 | 0.045 | 0.229 | 0.000 | 0.000 | 0.000 | 0.031 | 0.000 | 0.000 | 0.000 | 0.000 | 0.000 | 0.000 | 0.000 | 0.092 | 0.049 |
| Golden-crowned Sparrow | 0.106 | 0.047 | 0.000 | 0.000 | 0.000 | 0.000 | 0.000 | 0.000 | 0.000 | 0.000 | 0.000 | 0.000 | 0.061 | 0.000 | 0.000 | 0.000 | 0.000 | 0.199 | 0.057 | 0.133 | 0.000 | 0.398 | 0.000 | 0.000 | 0.000 |
| Gray Flycatcher | 0.000 | 0.000 | 0.000 | 0.000 | 0.000 | 0.000 | 0.000 | 0.000 | 0.467 | 0.000 | 0.000 | 0.000 | 0.000 | 0.000 | 0.000 | 0.000 | 0.000 | 0.000 | 0.533 | 0.000 | 0.000 | 0.000 | 0.000 | 0.000 | 0.000 |
| Gray Jay | 0.000 | 0.069 | 0.000 | 0.146 | 0.000 | 0.000 | 0.000 | 0.000 | 0.000 | 0.000 | 0.000 | 0.702 | 0.000 | 0.000 | 0.000 | 0.000 | 0.000 | 0.000 | 0.000 | 0.000 | 0.084 | 0.000 | 0.000 | 0.000 | 0.000 |
| Green-tailed Towhee | 0.000 | 0.000 | 0.000 | 0.000 | 0.000 | 0.000 | 0.000 | 0.000 | 0.000 | 0.000 | 0.000 | 0.000 | 0.000 | 0.000 | 0.000 | 0.000 | 0.000 | 0.000 | 0.000 | 0.000 | 1.000 | 0.000 | 0.000 | 0.000 | 0.000 |
| Hairy Woodpecker | 0.094 | 0.021 | 0.032 | 0.088 | 0.000 | 0.000 | 0.000 | 0.161 | 0.022 | 0.000 | 0.048 | 0.024 | 0.000 | 0.035 | 0.027 | 0.044 | 0.000 | 0.022 | 0.025 | 0.059 | 0.025 | 0.000 | 0.094 | 0.000 | 0.177 |
| Hammond's Flycatcher | 0.029 | 0.091 | 0.000 | 0.207 | 0.020 | 0.000 | 0.000 | 0.000 | 0.097 | 0.000 | 0.000 | 0.426 | 0.000 | 0.000 | 0.000 | 0.083 | 0.000 | 0.000 | 0.047 | 0.000 | 0.000 | 0.000 | 0.000 | 0.000 | 0.000 |
| Hermit Thrush | 0.033 | 0.000 | 0.000 | 0.278 | 0.000 | 0.017 | 0.023 | 0.034 | 0.008 | 0.062 | 0.006 | 0.165 | 0.029 | 0.000 | 0.000 | 0.162 | 0.008 | 0.000 | 0.053 | 0.000 | 0.062 | 0.062 | 0.000 | 0.000 | 0.000 |
| Hermit Warbler | 0.147 | 0.224 | 0.000 | 0.247 | 0.000 | 0.000 | 0.021 | 0.000 | 0.057 | 0.093 | 0.000 | 0.168 | 0.000 | 0.000 | 0.000 | 0.043 | 0.000 | 0.000 | 0.000 | 0.000 | 0.000 | 0.000 | 0.000 | 0.000 | 0.000 |
| House Finch | 0.000 | 0.000 | 0.021 | 0.000 | 0.000 | 0.000 | 0.000 | 0.000 | 0.000 | 0.000 | 0.191 | 0.000 | 0.000 | 0.000 | 0.000 | 0.000 | 0.000 | 0.000 | 0.000 | 0.039 | 0.000 | 0.702 | 0.000 | 0.000 | 0.047 |
| House Sparrow | 0.000 | 0.000 | 0.000 | 0.000 | 0.000 | 0.000 | 0.000 | 0.000 | 0.000 | 0.000 | 1.000 | 0.000 | 0.000 | 0.000 | 0.000 | 0.000 | 0.000 | 0.000 | 0.000 | 0.000 | 0.000 | 0.000 | 0.000 | 0.000 | 0.000 |
| House Wren | 0.288 | 0.169 | 0.000 | 0.000 | 0.000 | 0.000 | 0.000 | 0.000 | 0.000 | 0.080 | 0.000 | 0.000 | 0.000 | 0.000 | 0.000 | 0.270 | 0.000 | 0.090 | 0.000 | 0.000 | 0.103 | 0.000 | 0.000 | 0.000 | 0.000 |
| Hutton's Vireo | 0.000 | 0.000 | 0.000 | 0.000 | 0.075 | 0.055 | 0.225 | 0.000 | 0.000 | 0.138 | 0.188 | 0.000 | 0.000 | 0.000 | 0.000 | 0.000 | 0.000 | 0.000 | 0.000 | 0.000 | 0.000 | 0.000 | 0.000 | 0.155 | 0.165 |
| Lazuli Bunting | 0.000 | 0.000 | 0.000 | 0.000 | 0.000 | 0.000 | 0.000 | 0.000 | 0.000 | 0.345 | 0.000 | 0.000 | 0.239 | 0.000 | 0.000 | 0.194 | 0.000 | 0.000 | 0.222 | 0.000 | 0.000 | 0.000 | 0.000 | 0.000 | 0.000 |
| Lesser Goldfinch | 0.000 | 0.000 | 0.000 | 0.000 | 0.002 | 0.007 | 0.000 | 0.000 | 0.000 | 0.001 | 0.000 | 0.000 | 0.000 | 0.000 | 0.000 | 0.000 | 0.000 | 0.043 | 0.103 | 0.005 | 0.000 | 0.839 | 0.000 | 0.000 | 0.000 |
| Lincoln's Sparrow | 0.172 | 0.477 | 0.000 | 0.023 | 0.000 | 0.000 | 0.000 | 0.000 | 0.184 | 0.000 | 0.000 | 0.049 | 0.000 | 0.000 | 0.000 | 0.000 | 0.000 | 0.000 | 0.053 | 0.041 | 0.000 | 0.000 | 0.000 | 0.000 | 0.000 |
| MacGillivray's Warbler | 0.185 | 0.079 | 0.018 | 0.005 | 0.000 | 0.044 | 0.032 | 0.000 | 0.084 | 0.094 | 0.000 | 0.085 | 0.053 | 0.000 | 0.045 | 0.024 | 0.000 | 0.087 | 0.017 | 0.066 | 0.058 | 0.019 | 0.005 | 0.000 | 0.000 |
| Marsh Wren | 0.000 | 0.000 | 0.000 | 0.000 | 0.000 | 0.000 | 0.000 | 0.000 | 0.000 | 0.000 | 0.000 | 0.000 | 0.000 | 0.000 | 0.000 | 0.484 | 0.000 | 0.000 | 0.000 | 0.000 | 0.000 | 0.000 | 0.516 | 0.000 | 0.000 |
| Mountain Chickadee | 0.028 | 0.040 | 0.000 | 0.126 | 0.000 | 0.000 | 0.000 | 0.506 | 0.000 | 0.000 | 0.000 | 0.062 | 0.000 | 0.000 | 0.000 | 0.074 | 0.000 | 0.000 | 0.000 | 0.005 | 0.126 | 0.000 | 0.034 | 0.000 | 0.000 |
| Mourning Dove | 0.000 | 0.000 | 0.130 | 0.000 | 0.000 | 0.000 | 0.000 | 0.000 | 0.000 | 0.000 | 0.065 | 0.000 | 0.000 | 0.000 | 0.000 | 0.000 | 0.089 | 0.000 | 0.000 | 0.000 | 0.000 | 0.715 | 0.000 | 0.000 | 0.000 |
| Nashville Warbler | 0.235 | 0.047 | 0.000 | 0.223 | 0.000 | 0.000 | 0.016 | 0.058 | 0.050 | 0.054 | 0.000 | 0.077 | 0.000 | 0.000 | 0.000 | 0.101 | 0.000 | 0.029 | 0.021 | 0.003 | 0.074 | 0.000 | 0.012 | 0.000 | 0.000 |
| Northern Flicker | 0.000 | 0.066 | 0.000 | 0.056 | 0.020 | 0.030 | 0.000 | 0.081 | 0.000 | 0.012 | 0.182 | 0.059 | 0.000 | 0.000 | 0.000 | 0.014 | 0.000 | 0.028 | 0.048 | 0.037 | 0.032 | 0.223 | 0.089 | 0.000 | 0.022 |
| Orange-crowned Warbler | 0.160 | 0.149 | 0.017 | 0.086 | 0.014 | 0.004 | 0.000 | 0.030 | 0.053 | 0.027 | 0.021 | 0.081 | 0.007 | 0.000 | 0.000 | 0.032 | 0.006 | 0.002 | 0.007 | 0.108 | 0.107 | 0.046 | 0.018 | 0.002 | 0.024 |
| Pacific-slope/Cordilleran Flycatcher | 0.000 | 0.000 | 0.000 | 0.000 | 0.241 | 0.000 | 0.000 | 0.241 | 0.000 | 0.000 | 0.000 | 0.000 | 0.204 | 0.000 | 0.000 | 0.000 | 0.166 | 0.000 | 0.000 | 0.147 | 0.000 | 0.000 | 0.000 | 0.000 | 0.000 |
| Pacific Wren | 0.000 | 0.000 | 0.000 | 0.000 | 0.000 | 0.000 | 0.000 | 0.000 | 0.000 | 0.000 | 0.283 | 0.000 | 0.000 | 0.249 | 0.000 | 0.000 | 0.312 | 0.000 | 0.000 | 0.000 | 0.000 | 0.000 | 0.000 | 0.156 | 0.000 |
| Pileated Woodpecker | 0.000 | 0.000 | 0.000 | 0.000 | 0.000 | 0.000 | 0.000 | 0.000 | 0.000 | 0.000 | 0.000 | 1.000 | 0.000 | 0.000 | 0.000 | 0.000 | 0.000 | 0.000 | 0.000 | 0.000 | 0.000 | 0.000 | 0.000 | 0.000 | 0.000 |
| Pine Siskin | 0.034 | 0.037 | 0.000 | 0.024 | 0.000 | 0.000 | 0.000 | 0.000 | 0.040 | 0.000 | 0.144 | 0.042 | 0.000 | 0.000 | 0.000 | 0.016 | 0.000 | 0.000 | 0.027 | 0.000 | 0.000 | 0.635 | 0.000 | 0.000 | 0.000 |
| Purple Finch | 0.002 | 0.025 | 0.007 | 0.006 | 0.007 | 0.002 | 0.001 | 0.003 | 0.008 | 0.113 | 0.040 | 0.022 | 0.000 | 0.005 | 0.005 | 0.079 | 0.003 | 0.047 | 0.263 | 0.016 | 0.053 | 0.287 | 0.006 | 0.000 | 0.002 |
| Pygmy Nuthatch | 0.000 | 0.000 | 0.000 | 0.000 | 0.000 | 0.000 | 0.000 | 0.000 | 0.000 | 0.000 | 0.000 | 0.000 | 0.000 | 0.000 | 0.000 | 0.467 | 0.000 | 0.000 | 0.000 | 0.000 | 0.533 | 0.000 | 0.000 | 0.000 | 0.000 |
| Red Crossbill | 0.000 | 0.000 | 0.000 | 0.000 | 0.000 | 0.000 | 0.000 | 0.000 | 0.186 | 0.000 | 0.814 | 0.000 | 0.000 | 0.000 | 0.000 | 0.000 | 0.000 | 0.000 | 0.000 | 0.000 | 0.000 | 0.000 | 0.000 | 0.000 | 0.000 |
| Red-breasted Nuthatch | 0.120 | 0.148 | 0.000 | 0.348 | 0.000 | 0.000 | 0.000 | 0.082 | 0.022 | 0.000 | 0.008 | 0.215 | 0.000 | 0.000 | 0.000 | 0.045 | 0.000 | 0.000 | 0.000 | 0.000 | 0.013 | 0.000 | 0.000 | 0.000 | 0.000 |
| Red-breasted Sapsucker | 0.133 | 0.102 | 0.000 | 0.087 | 0.008 | 0.000 | 0.000 | 0.197 | 0.098 | 0.024 | 0.000 | 0.035 | 0.000 | 0.000 | 0.000 | 0.141 | 0.000 | 0.022 | 0.050 | 0.043 | 0.056 | 0.000 | 0.006 | 0.000 | 0.000 |
| Red-naped Sapsucker | 0.423 | 0.000 | 0.000 | 0.000 | 0.000 | 0.000 | 0.000 | 0.577 | 0.000 | 0.000 | 0.000 | 0.000 | 0.000 | 0.000 | 0.000 | 0.000 | 0.000 | 0.000 | 0.000 | 0.000 | 0.000 | 0.000 | 0.000 | 0.000 | 0.000 |
| Red-winged Blackbird | 0.000 | 0.000 | 0.000 | 0.100 | 0.000 | 0.000 | 0.000 | 0.000 | 0.000 | 0.000 | 0.000 | 0.000 | 0.000 | 0.000 | 0.000 | 0.093 | 0.000 | 0.000 | 0.000 | 0.000 | 0.000 | 0.744 | 0.064 | 0.000 | 0.000 |
| Ruby-crowned Kinglet | 0.000 | 0.000 | 0.000 | 0.284 | 0.000 | 0.000 | 0.000 | 0.413 | 0.000 | 0.000 | 0.000 | 0.303 | 0.000 | 0.000 | 0.000 | 0.000 | 0.000 | 0.000 | 0.000 | 0.000 | 0.000 | 0.000 | 0.000 | 0.000 | 0.000 |
| Rufous Hummingbird | 0.262 | 0.231 | 0.000 | 0.000 | 0.000 | 0.000 | 0.000 | 0.000 | 0.000 | 0.000 | 0.000 | 0.262 | 0.000 | 0.000 | 0.000 | 0.000 | 0.245 | 0.000 | 0.000 | 0.000 | 0.000 | 0.000 | 0.000 | 0.000 | 0.000 |
| Song Sparrow | 0.007 | 0.013 | 0.024 | 0.040 | 0.014 | 0.009 | 0.001 | 0.006 | 0.044 | 0.016 | 0.065 | 0.012 | 0.028 | 0.031 | 0.026 | 0.029 | 0.093 | 0.023 | 0.023 | 0.153 | 0.064 | 0.161 | 0.070 | 0.009 | 0.040 |
| Spotted Towhee | 0.006 | 0.003 | 0.040 | 0.011 | 0.036 | 0.070 | 0.000 | 0.052 | 0.000 | 0.034 | 0.000 | 0.000 | 0.044 | 0.000 | 0.000 | 0.076 | 0.003 | 0.082 | 0.062 | 0.255 | 0.031 | 0.197 | 0.000 | 0.000 | 0.000 |
| Steller's Jay | 0.029 | 0.043 | 0.000 | 0.083 | 0.013 | 0.138 | 0.134 | 0.040 | 0.055 | 0.057 | 0.000 | 0.010 | 0.011 | 0.000 | 0.034 | 0.037 | 0.000 | 0.000 | 0.095 | 0.016 | 0.074 | 0.000 | 0.039 | 0.046 | 0.044 |
| Swainson's Thrush | 0.000 | 0.004 | 0.040 | 0.004 | 0.104 | 0.043 | 0.006 | 0.000 | 0.006 | 0.039 | 0.066 | 0.016 | 0.085 | 0.077 | 0.096 | 0.019 | 0.078 | 0.031 | 0.000 | 0.164 | 0.000 | 0.034 | 0.009 | 0.050 | 0.030 |
| Townsend's Solitaire | 0.000 | 0.000 | 0.000 | 0.062 | 0.000 | 0.000 | 0.000 | 0.538 | 0.000 | 0.000 | 0.000 | 0.066 | 0.000 | 0.000 | 0.000 | 0.123 | 0.000 | 0.000 | 0.000 | 0.000 | 0.211 | 0.000 | 0.000 | 0.000 | 0.000 |
| Townsend's Warbler | 0.000 | 0.000 | 0.000 | 0.000 | 0.000 | 0.000 | 0.000 | 0.000 | 0.000 | 0.000 | 0.000 | 0.348 | 0.000 | 0.000 | 0.000 | 0.652 | 0.000 | 0.000 | 0.000 | 0.000 | 0.000 | 0.000 | 0.000 | 0.000 | 0.000 |
| Varied Thrush | 0.000 | 0.000 | 0.000 | 0.000 | 0.000 | 0.000 | 0.000 | 0.000 | 0.500 | 0.000 | 0.000 | 0.000 | 0.000 | 0.000 | 0.000 | 0.000 | 0.500 | 0.000 | 0.000 | 0.000 | 0.000 | 0.000 | 0.000 | 0.000 | 0.000 |
| Vesper Sparrow | 1.000 | 0.000 | 0.000 | 0.000 | 0.000 | 0.000 | 0.000 | 0.000 | 0.000 | 0.000 | 0.000 | 0.000 | 0.000 | 0.000 | 0.000 | 0.000 | 0.000 | 0.000 | 0.000 | 0.000 | 0.000 | 0.000 | 0.000 | 0.000 | 0.000 |
| Warbling Vireo | 0.000 | 0.125 | 0.000 | 0.000 | 0.096 | 0.000 | 0.000 | 0.000 | 0.000 | 0.000 | 0.000 | 0.000 | 0.000 | 0.106 | 0.000 | 0.066 | 0.000 | 0.000 | 0.606 | 0.000 | 0.000 | 0.000 | 0.000 | 0.000 | 0.000 |
| Western Bluebird | 0.000 | 0.000 | 0.000 | 0.000 | 0.000 | 0.000 | 0.000 | 0.000 | 0.000 | 0.000 | 0.000 | 0.000 | 0.000 | 0.000 | 0.000 | 0.000 | 0.000 | 0.000 | 1.000 | 0.000 | 0.000 | 0.000 | 0.000 | 0.000 | 0.000 |
| Western Scrub-Jay | 0.000 | 0.000 | 0.147 | 0.000 | 0.147 | 0.054 | 0.000 | 0.000 | 0.000 | 0.000 | 0.000 | 0.000 | 0.016 | 0.000 | 0.000 | 0.000 | 0.000 | 0.051 | 0.029 | 0.023 | 0.000 | 0.507 | 0.027 | 0.000 | 0.000 |
| Western Tanager | 0.018 | 0.000 | 0.075 | 0.103 | 0.138 | 0.083 | 0.000 | 0.013 | 0.000 | 0.008 | 0.006 | 0.000 | 0.064 | 0.000 | 0.021 | 0.026 | 0.000 | 0.034 | 0.039 | 0.107 | 0.059 | 0.206 | 0.000 | 0.000 | 0.000 |
| White-breasted Nuthatch | 0.000 | 0.000 | 0.000 | 0.000 | 0.000 | 0.000 | 0.000 | 0.000 | 0.000 | 0.000 | 0.000 | 0.000 | 0.000 | 0.000 | 0.000 | 0.127 | 0.000 | 0.000 | 0.291 | 0.000 | 0.073 | 0.509 | 0.000 | 0.000 | 0.000 |
| White-crowned Sparrow | 0.000 | 0.000 | 0.000 | 0.000 | 0.000 | 0.043 | 0.000 | 0.020 | 0.000 | 0.024 | 0.617 | 0.000 | 0.000 | 0.000 | 0.000 | 0.000 | 0.269 | 0.000 | 0.015 | 0.012 | 0.000 | 0.000 | 0.000 | 0.000 | 0.000 |
| White-headed Woodpecker | 0.000 | 0.000 | 0.000 | 0.467 | 0.000 | 0.000 | 0.000 | 0.000 | 0.000 | 0.000 | 0.000 | 0.000 | 0.000 | 0.000 | 0.000 | 0.000 | 0.000 | 0.000 | 0.533 | 0.000 | 0.000 | 0.000 | 0.000 | 0.000 | 0.000 |
| Williamson's Sapsucker | 0.000 | 0.000 | 0.000 | 0.000 | 0.000 | 0.000 | 0.000 | 0.000 | 0.000 | 0.000 | 0.000 | 0.000 | 0.000 | 0.000 | 0.000 | 1.000 | 0.000 | 0.000 | 0.000 | 0.000 | 0.000 | 0.000 | 0.000 | 0.000 | 0.000 |
| Willow Flycatcher | 0.000 | 0.000 | 0.000 | 0.229 | 0.000 | 0.000 | 0.000 | 0.000 | 0.000 | 0.000 | 0.000 | 0.244 | 0.282 | 0.000 | 0.000 | 0.000 | 0.000 | 0.000 | 0.000 | 0.000 | 0.000 | 0.000 | 0.244 | 0.000 | 0.000 |
| Wilson's Warbler | 0.052 | 0.235 | 0.009 | 0.010 | 0.000 | 0.007 | 0.005 | 0.000 | 0.074 | 0.014 | 0.024 | 0.114 | 0.096 | 0.057 | 0.076 | 0.006 | 0.019 | 0.032 | 0.015 | 0.003 | 0.000 | 0.000 | 0.024 | 0.029 | 0.098 |
| Wrentit | 0.000 | 0.000 | 0.025 | 0.000 | 0.087 | 0.072 | 0.000 | 0.000 | 0.000 | 0.000 | 0.160 | 0.000 | 0.080 | 0.044 | 0.102 | 0.000 | 0.130 | 0.005 | 0.000 | 0.140 | 0.000 | 0.080 | 0.000 | 0.025 | 0.048 |
| Yellow Warbler | 0.009 | 0.011 | 0.059 | 0.073 | 0.008 | 0.003 | 0.000 | 0.000 | 0.000 | 0.000 | 0.000 | 0.000 | 0.061 | 0.019 | 0.000 | 0.087 | 0.000 | 0.148 | 0.275 | 0.052 | 0.043 | 0.046 | 0.105 | 0.000 | 0.000 |
| Yellow-breasted Chat | 0.000 | 0.000 | 0.072 | 0.000 | 0.157 | 0.053 | 0.000 | 0.000 | 0.000 | 0.000 | 0.000 | 0.000 | 0.112 | 0.007 | 0.015 | 0.000 | 0.000 | 0.174 | 0.024 | 0.180 | 0.000 | 0.199 | 0.000 | 0.000 | 0.007 |
| Yellow-rumped Warbler | 0.148 | 0.094 | 0.000 | 0.230 | 0.000 | 0.000 | 0.000 | 0.025 | 0.007 | 0.000 | 0.019 | 0.081 | 0.003 | 0.000 | 0.000 | 0.041 | 0.000 | 0.000 | 0.047 | 0.000 | 0.278 | 0.000 | 0.026 | 0.000 | 0.000 |

Appendix 2. Results from the Chao’s abundance-based Jaccard community similarity analysis assessing breeding, molting, and naïve bird communities at the 25 capture stations in southern Oregon and Northern California

Appendix 2, Figure 1. Results from the Chao’s abundance-based Jaccard community similarity analysis assessing breeding bird communities at the 25 capture stations (z-transformed values and arranged by elevation). Lower (-) and higher (+) values represent dissimilar and similar bird communities, respectively.

Appendix 2, Figure 2. Results from the Chao’s abundance-based Jaccard community similarity analysis assessing molting bird communities at the 25 capture stations (z-transformed values and arranged by elevation). Lower (-) and higher (+) values represent dissimilar and similar bird communities, respectively.

Appendix 2, Figure 3. Results from the Chao’s abundance-based Jaccard community similarity analysis assessing naïve bird communities at the 25 capture stations (z-transformed values and arranged by elevation). Lower (-) and higher (+) values represent dissimilar and similar bird communities, respectively.

Appendix 3. Detrended Correspondence Analysis (DCA) eigenvalues and axis lengths.

|  | *Naïve Community* | | | |
| --- | --- | --- | --- | --- |
|  | DCA1 | DCA2 | DCA3 | DCA4 |
| Eigenvalues | 0.33 | 0.26 | 0.15 | 0.12 |
| Axis lengths | 3.57 | 3.70 | 4.28 | 3.95 |
|  |  |  |  |  |
|  | *Breeding Community* | | | |
|  | DCA1 | DCA2 | DCA3 | DCA4 |
| Eigenvalues | 0.41 | 0.33 | 0.23 | 0.14 |
| Axis lengths | 4.99 | 3.74 | 4.32 | 4.07 |
|  |  |  |  |  |
|  | *Molting Community* | | | |
|  | DCA1 | DCA2 | DCA3 | DCA4 |
| Eigenvalues | 0.62 | 0.32 | 0.21 | 0.16 |
| Axis lengths | 4.41 | 4.16 | 2.98 | 3.63 |

Appendix 4. Stations and their direction cosines of the vectors used in the ordination, squared correlation coeﬃcient (r^2^), and respective p-values for birds captured between May through October.

|  | Naïve | | | |  | Breeding | | | |  | Molting | | | |
| --- | --- | --- | --- | --- | --- | --- | --- | --- | --- | --- | --- | --- | --- | --- |
| Station | DCA1 | DCA2 | r^2^ | Pr(>r) |  | DCA1 | DCA2 | r^2^ | Pr(>r) |  | DCA1 | DCA2 | r^2^ | Pr(>r) |
| 7MIL | 0.993 | -0.117 | 0.353 | 0.009** |  | 0.929 | -0.370 | 0.236 | 0.043* |  | 0.994 | -0.109 | 0.480 | 0.000*** |
| ANT1 | 0.982 | -0.190 | 0.200 | 0.061 |  | 0.994 | 0.114 | 0.232 | 0.055 |  | 0.984 | -0.179 | 0.485 | 0.000*** |
| APRI | -0.854 | 0.521 | 0.045 | 0.328 |  | -0.715 | -0.699 | 0.114 | 0.157 |  | -0.181 | 0.984 | 0.136 | 0.210 |
| CABN | 0.465 | -0.885 | 0.106 | 0.162 |  | -0.468 | -0.883 | 0.151 | 0.113 |  | 0.979 | -0.203 | 0.429 | 0.000*** |
| CAMP | -0.443 | 0.896 | 0.528 | 0.000*** |  | -0.637 | 0.771 | 0.214 | 0.073 |  | -0.178 | 0.984 | 0.277 | 0.026* |
| CAPD | -0.352 | 0.936 | 0.185 | 0.080 |  | -0.965 | 0.260 | 0.002 | 0.971 |  | -0.108 | 0.994 | 0.326 | 0.009** |
| GBCR | 0.470 | 0.883 | 0.361 | 0.015* |  | 0.686 | 0.728 | 0.326 | 0.026* |  | 1.000 | 0.022 | 0.299 | 0.000 *** |
| GERB | 0.979 | -0.203 | 0.372 | 0.014* |  | 0.644 | -0.765 | 0.143 | 0.115 |  | 0.960 | -0.279 | 0.377 | 0.000*** |
| GROV | 1.000 | 0.025 | 0.109 | 0.122 |  | 0.550 | -0.835 | 0.033 | 0.542 |  | 0.986 | 0.169 | 0.303 | 0.005** |
| HCME | 0.905 | 0.426 | 0.102 | 0.119 |  | 0.952 | -0.305 | 0.060 | 0.365 |  | 0.145 | -0.989 | 0.128 | 0.169 |
| HOME | -0.623 | 0.782 | 0.254 | 0.029* |  | -0.594 | 0.804 | 0.248 | 0.053 |  | -0.614 | 0.790 | 0.321 | 0.007** |
| JOHN | 0.998 | 0.067 | 0.394 | 0.006** |  | 0.999 | -0.039 | 0.428 | 0.009** |  | 0.992 | -0.126 | 0.393 | 0.000*** |
| LADY | -0.509 | 0.861 | 0.276 | 0.031* |  | -0.892 | 0.451 | 0.100 | 0.200 |  | -0.080 | 0.997 | 0.441 | 0.000*** |
| LELA | -0.480 | 0.877 | 0.415 | 0.011* |  | -0.454 | 0.891 | 0.351 | 0.022* |  | -0.178 | 0.984 | 0.416 | 0.000*** |
| MARI | -0.383 | 0.924 | 0.439 | 0.007** |  | -0.346 | 0.938 | 0.316 | 0.026* |  | -0.086 | 0.996 | 0.508 | 0.000*** |
| ODES | -0.207 | -0.978 | 0.014 | 0.693 |  | -0.491 | -0.871 | 0.123 | 0.147 |  | 0.400 | -0.917 | 0.221 | 0.076 |
| PARK | -0.550 | 0.835 | 0.305 | 0.026* |  | -0.664 | 0.748 | 0.197 | 0.064 |  | -0.174 | 0.985 | 0.316 | 0.004** |
| PCT1 | -0.832 | 0.554 | 0.033 | 0.455 |  | -0.339 | -0.941 | 0.084 | 0.226 |  | -0.393 | -0.919 | 0.148 | 0.170 |
| TOPS | 0.279 | -0.960 | 0.010 | 0.754 |  | -0.255 | -0.967 | 0.027 | 0.538 |  | -0.119 | -0.993 | 0.316 | 0.005** |
| WIIM | -0.764 | 0.645 | 0.066 | 0.237 |  | -0.894 | -0.449 | 0.070 | 0.259 |  | -0.130 | 0.991 | 0.220 | 0.067 |
| WILL | 0.867 | -0.499 | 0.023 | 0.581 |  | -0.331 | -0.944 | 0.050 | 0.394 |  | 0.761 | -0.649 | 0.254 | 0.042* |
| WIWI | -0.839 | -0.544 | 0.374 | 0.017* |  | -0.802 | -0.597 | 0.282 | 0.026* |  | -0.848 | -0.530 | 0.511 | 0.000*** |
| WOOD | -0.763 | -0.647 | 0.087 | 0.178 |  | -0.690 | -0.723 | 0.147 | 0.085 |  | -0.302 | 0.953 | 0.047 | 0.478 |
| WREF | -0.408 | 0.913 | 0.493 | 0.000*** |  | -0.328 | 0.945 | 0.513 | 0.000*** |  | -0.079 | 0.997 | 0.491 | 0.000*** |
| YACR | -0.443 | 0.896 | 0.322 | 0.025* |  | -0.541 | 0.841 | 0.205 | 0.052 |  | 0.074 | 0.997 | 0.339 | 0.005** |

Appendix 5. Detrended Correspondence Analysis (DCA) ordination for breeding, molting, and naïve bird communities from southern Oregon and Northern California

Appendix 5, Figure 1. Detrended Correspondence Analysis (DCA) ordination for breeding bird communities denoted with convex hull polygons encompassing regions with more than five banding stations. Data used in the DCA came from captured individuals from May-October.

Appendix 5, Figure 2. Detrended Correspondence Analysis (DCA) ordination for molting bird communities denoted with convex hull polygons encompassing regions with more than five banding stations. Data used in the DCA came from captured individuals from May-October.

Appendix 5, Figure 3. Detrended Correspondence Analysis (DCA) ordination for naïve bird communities denoted with convex hull polygons encompassing regions with more than five banding stations. Data used in the DCA came from captured individuals from May-October.

Appendix 6. Species diversity statistics for the 25 capture stations in northern California and Southern Oregon.

|  | Naïve | | | Breeding | | | Molting | | |
| --- | --- | --- | --- | --- | --- | --- | --- | --- | --- |
| Station | Rarefaction (extrapolated 40 years) | Chao I (10-year) | Shannon (10-year ) | Rarefaction (extrapolated 40 years) | Chao I (10-year) | Shannon (10-year) | Rarefaction (extrapolated 40 years) | Chao I (10-year) | Shannon (10-year ) |
| 7MIL | 71.44 | 66.55 | 3.28 | 55.27 | 50.27 | 2.82 | 43.93 | 40.62 | 2.50 |
| ANT1 | 64.29 | 56.31 | 3.14 | 50.13 | 47.45 | 2.75 | 39.02 | 40.09 | 2.74 |
| APRI | 52.80 | 54.09 | 3.15 | 35.71 | 32.41 | 2.64 | 27.07 | 23.69 | 2.76 |
| CABN | 80.35 | 72.62 | 3.41 | 59.20 | 54.54 | 2.79 | 45.16 | 43.89 | 2.94 |
| CAMP | 57.97 | 59.85 | 3.05 | 42.88 | 33.86 | 2.41 | 46.69 | 48.15 | 2.67 |
| CAPD | 63.78 | 57.35 | 3.17 | 47.99 | 43.10 | 2.61 | 29.60 | 26.50 | 2.73 |
| GBCR | 48.56 | 51.41 | 2.66 | 25.36 | 24.92 | 2.38 | 24.64 | 21.46 | 2.48 |
| GERB | 57.71 | 56.85 | 3.19 | 47.04 | 49.58 | 2.79 | 33.33 | 33.8 | 2.51 |
| GROV | 69.81 | 65.12 | 3.08 | 53.63 | 38.75 | 2.88 | 37.56 | 27.97 | 2.16 |
| HCME | 69.36 | 60.53 | 3.08 | 44.85 | 41.55 | 2.82 | 44.36 | 32.68 | 2.52 |
| HOME | 69.43 | 67.39 | 3.17 | 57.11 | 54.17 | 2.58 | 40.64 | 34.19 | 2.83 |
| JOHN | 60.27 | 62.99 | 3.12 | 46.23 | 41.98 | 2.72 | 46.23 | 41.98 | 2.72 |
| LADY | 53.68 | 55.28 | 2.94 | 41.11 | 34.56 | 2.41 | 33.17 | 35.84 | 2.53 |
| LELA | 59.04 | 51.00 | 2.48 | 30.45 | 31.00 | 2.13 | 19.79 | 20.30 | 2.33 |
| MARI | 43.67 | 41.12 | 2.42 | 30.55 | 28.06 | 2.14 | 15.83 | 16.12 | 2.06 |
| ODES | 74.90 | 71.99 | 3.36 | 54.67 | 53.69 | 2.85 | 50.46 | 45.58 | 3.08 |
| PARK | 59.11 | 53.11 | 2.66 | 34.76 | 30.01 | 2.20 | 34.11 | 29.08 | 2.09 |
| PCT1 | 76.93 | 68.95 | 3.16 | 48.56 | 44.29 | 2.61 | 36.76 | 36.06 | 2.80 |
| TOPS | 78.15 | 76.00 | 2.95 | 69.34 | 58.12 | 2.50 | 53.48 | 51.75 | 2.34 |
| WIIM | 70.45 | 64.96 | 3.03 | 48.12 | 49.12 | 2.47 | 35.29 | 36.09 | 2.62 |
| WILL | 64.29 | 65.2 | 3.24 | 53.32 | 49.12 | 2.75 | 41.06 | 35.41 | 2.79 |
| WIWI | 82.52 | 75.96 | 3.25 | 45.18 | 43.31 | 2.51 | 41.71 | 41.57 | 2.31 |
| WOOD | 67.5 | 67.06 | 2.74 | 45.44 | 45.19 | 1.98 | 35.23 | 30.57 | 2.56 |
| WREF | 43.36 | 36.53 | 2.24 | 29.09 | 26.31 | 2.03 | 16.36 | 11.18 | 1.98 |
| YACR | 56.98 | 59.5 | 2.73 | 42.14 | 42.75 | 2.47 | 32.56 | 27.95 | 2.57 |
